# Mesenchymal Stromal Cells Epithelial Transition Induced by Renal Tubular Cells-Derived Extracellular Vesicles

**DOI:** 10.1371/journal.pone.0159163

**Published:** 2016-07-13

**Authors:** Giulia Chiabotto, Stefania Bruno, Federica Collino, Giovanni Camussi

**Affiliations:** 1 Department of Medical Science, University of Torino, Medical School, Torino, Italy; 2 Department of Molecular Biotechnology and Healthy Science, Molecular Biotechnology Center, University of Torino, Torino, Italy; Center for Molecular Biotechnology, ITALY

## Abstract

Mesenchymal-epithelial interactions play an important role in renal tubular morphogenesis and in maintaining the structure of the kidney. The aim of this study was to investigate whether extracellular vesicles (EVs) produced by human renal proximal tubular epithelial cells (RPTECs) may induce mesenchymal-epithelial transition of bone marrow-derived mesenchymal stromal cells (MSCs). To test this hypothesis, we characterized the phenotype and the RNA content of EVs and we evaluated the *in vitro* uptake and activity of EVs on MSCs. MicroRNA (miRNA) analysis suggested the possible implication of the miR-200 family carried by EVs in the epithelial commitment of MSCs. Bone marrow-derived MSCs were incubated with EVs, or RPTEC-derived total conditioned medium, or conditioned medium depleted of EVs. As a positive control, MSCs were co-cultured in a transwell system with RPTECs. Epithelial commitment of MSCs was assessed by real time PCR and by immunofluorescence analysis of cellular expression of specific mesenchymal and epithelial markers. After one week of incubation with EVs and total conditioned medium, we observed mesenchymal-epithelial transition in MSCs. Stimulation with conditioned medium depleted of EVs did not induce any change in mesenchymal and epithelial gene expression. Since EVs were found to contain the miR-200 family, we transfected MSCs using synthetic miR-200 mimics. After one week of transfection, mesenchymal-epithelial transition was induced in MSCs. In conclusion, miR-200 carrying EVs released from RPTECs induce the epithelial commitment of MSCs that may contribute to their regenerative potential. Based on experiments of MSC transfection with miR-200 mimics, we suggested that the miR-200 family may be involved in mesenchymal-epithelial transition of MSCs.

## Introduction

Mutual interactions between epithelial cells and mesenchymal cells coordinate kidney development, play a pivotal role in maintaining organ integrity in the adult, and contribute to renal regeneration after injury. Bone marrow-derived mesenchymal stromal cells (MSCs) have multipotent characteristics, since they can differentiate into adipocytes, osteocytes and chondrocytes. Moreover, the epithelial commitment of bone marrow-derived MSCs induced by renal tubular epithelial cells has been demonstrated in co-culture conditions [1]. Recently, conditioned medium derived from renal tubular epithelial cells has also proved to induce an epithelial commitment of adipose-derived adult MSCs [2] and of bone marrow-derived MSCs [3,4].

The epithelial reprogramming of MSCs consists in the acquisition of morphological, antigenic and functional properties of polarized epithelial cells. Mesenchymal-epithelial transition (MET) has been defined as an activation of epithelial genes, including those encoding for cytokeratins, desmosomes, adherens and tight junctions, and an inactivation of mesenchymal genes, such as vimentin and collagen [5]. MET is a phenomenon observed during nephrogenesis, when the metanephric mesenchyme develops into nephrons [6]. During embryogenesis, both MET and epithelial-mesenchymal transition (EMT)—the reversed program of MET—are essential for organ development. While there are plenty of studies analyzing EMT related to fibrosis in chronic inflammation and metastasis of tumor [7–13], relatively little is known about MET associated with kidney formation. This process seems to be regulated by genes such as paired box 2 (*PAX2*), bone morphogenetic protein 7 (*BMP7*), and Wilms tumor 1 (*WT1*) [14–16].

Besides physical interactions, such as direct cell-to-cell contact, nanotubes and cytonemes, and the release of soluble factors, the extracellular vesicles (EVs) have recently emerged as an important mechanism of communication between cells. The term EVs includes exosomes, originating from the membrane of the endosomal compartment, and microvesicles, derived from plasma membrane budding. Both of them are small membrane fragments that can shuttle cytosolic proteins, receptors, bioactive lipids and nucleic acids to target cells, acting as a potent paracrine mechanism that can re-direct cell fate [17]. Ratajczak *et al*.[18] first described that EVs derived from embryonic stem cells may reprogram hematopoietic progenitors by a mRNA-dependent mechanism. Quesenberry and Aliotta suggested that a continuous genetic modulation of cells through transfer of EVs may be involved in the continuum change in bone marrow stem cell phenotype. EV-mediated transfer of proteins and genetic information from injured cells to bone marrow-derived stem cells may reprogram their phenotype to acquire features of the injured tissue [19,20].

EVs carry proteins, lipids, and nucleic acids, including microRNAs (miRNAs) [21–23] short non-coding RNAs that regulate post-transcriptional expression of several genes, either by triggering mRNA cleavage or by repressing translation [24,25].

The aim of the present study was to evaluate whether renal tubular epithelial cell (RPTEC)-derived EVs are involved in the epithelial differentiation of bone marrow-derived MSCs and the role of miRNAs carried by EVs.

## Materials and Methods

### Cell culture

Human bone marrow-derived MSCs were obtained from Lonza (Basel, Switzerland), cultured and characterized as previously described [26,27]. Briefly, MSCs were cultured in the presence of Mesenchymal Stem Cells Basal Medium (MSCBM, Lonza) and maintained in an incubator with a humidified atmosphere of 5% CO_2_ at 37°C. MSCs were seeded at a density of 10,000 cells/cm^2^ and used within the seventh passage. The adipogenic, osteogenic and chondrogenic differentiation ability of MSCs was determined as previously described [26,27].

Human RPTECs were obtained from Lonza and cultured in Renal Epithelial Cell Basal Medium (REBM, Lonza) supplemented with Renal Epithelial Cell Growth Medium Bullet Kit (REGM, Lonza), according to the manufacturer’s instructions.

### Immunofluorescence analyses

Indirect immunofluorescence was performed on cells cultured on chamber slides (Nalgen Nunc International, Rochester, NY, USA). The cells were fixed in 2,5% paraformaldehyde and permeabilized with Hepes-Triton X100 buffer (Sigma-Aldrich, St. Louis, MO). The following antibodies were used: mouse monoclonal anti-vimentin (Sigma-Aldrich), mouse monoclonal anti-pan-cytokeratin (Bio-Rad, Hercules, CA), rabbit polyclonal anti-cytokeratin 18 and goat polyclonal anti-aminopeptidase A (Santa Cruz Biotechnology, Dallas, TX). As a control, primary antibodies were omitted and substituted with nonimmune mouse IgG. Alexa Fluor 488 anti-mouse (Invitrogen, Carlsbad, CA) was used as secondary antibody. For nuclear staining, Hoechst 33258 dye (Sigma-Aldrich) was added to fixed cells. A Zeiss LSM 5 Pascal Model Confocal Microscope (Carl Zeiss International, Germany) was used to perform confocal microscopy analysis.

MSCs expressed high levels of vimentin (S1A Fig), while cytokeratins expression was not detected (S1B Fig). RPTECs expressed low levels of vimentin (S2A Fig) and high levels of cytokeratins (S2B Fig).

### Cytofluorimetric analyses

Cells were counted at each passage and their immunophenotype was analyzed by cytofluorimetric analysis, using FACS Calibur (BD Biosciences, San Jose, CA). The following antibodies, all fluorescein isothiocyanate (FITC) or phycoerythrin (PE) conjugated were used: anti-CD105, -CD146 (Miltenyi Biotech, Bergisch Gladbach, Germany); -CD29, -CD44, -CD45, -alpha5 integrin (CD49e), -alpha6 integrin (CD49f), -CD73, -CD90, -VEGFR2 (Becton Dickinson Biosciences Pharmingen, San Jose, CA); -EpCAM, -HLA-I (BioLegend, San Diego, CA). Mouse IgG isotypic controls were from Miltenyi.

MSCs showed the expression of typical MSC surface marker molecules: CD29 (integrin beta-1), CD44, CD73 (ecto-5’-nucleotidase), CD90 (Thy-1 cell surface antigen), CD105 (endoglin) and CD146 (melanoma cell adhesion molecule, MCAM), while epithelial marker EpCAM was not detected (S1C Fig).

RPTECs showed the expression of CD24, CD29, CD44, alpha-5 integrin (CD49e), alpha-6 integrin (CD49f), CD73, CD146, EpCAM, and HLA-class I surface molecules while hematopoietic marker CD45, endothelial marker VEGFR2, and mesenchymal stromal cell marker CD105 were not detected (S2C Fig).

### Extracellular vesicles isolation and conditioned medium preparation

EVs were obtained from supernatant of ≥80% confluent RPTECs cultured overnight in RPMI (without phenol red, Invitrogen) supplemented with 2% FBS deprived of EVs after 15 hours of centrifugation at 100,000 g (Optima L-90K ultracentrifuge, Beckman Coulter, Indianapolis, IN). Cell supernatant was collected, centrifuged at 2,000 g for 20 minutes to remove debris, and processed to obtain: (1) extracellular vesicles (EVs), (2) total conditioned medium with EVs (TOT-CM) and (3) conditioned medium after EVs deprivation (CM) after ultracentrifugation at 100,000 g for 15 hours.

EVs were isolated from cell-free supernatant by differential ultracentrifugation as previously described by Théry and colleagues [28]. The pellet collected after ultracentrifugation at 100,000 g was resuspended in RPMI containing 10% dimethyl sulfoxide (DMSO, Sigma-Aldrich) and stored at -80°C.

Particles size and concentration were measured by NanoSight LM10 instrument (NanoSight Ltd, Amesbury, UK) equipped with the nanoparticle tracking analyses (NTA) 2.0 analytic software [29,30].

TOT-CM and CM were concentrated ~20-fold by centrifugation at 2,700 g for 40 minutes at 4°C, using ultrafiltration units with a 3 kDa molecular weight cut-off (Amicon Ultra -15, centrifugal filter devices, Millipore, Billerica, MA). To preserve EVs integrity, TOT-CM was supplemented with 10% DMSO and stored at -80°C, while CM was stored directly at -20°C.

### EV characterization

Cytofluorimetric analysis was performed as previously described [30,31]. Briefly, EVs were incubated for 15 minutes at 4°C with the following FITC- or PE-conjugated antibodies: anti-CD24, -CD29, -CD44, -alpha5 integrin (CD49e), -alpha6 integrin (CD49f), -CD63, -CD73, -CD81, -CD107, (Becton Dickinson), -CD146, -EpCAM, -HLA1 (BioLegend, San Diego, CA). FITC or PE mouse nonimmune isotypic IgG (Miltenyi) were used as control. For each EV preparation, 5,000 particles were acquired using Guava easyCyte™ Flow Cytometer (Millipore) and analysed with the InCyte™ software.

### Incorporation of EVs in MSCs

To study EVs incorporation by MSCs, we incubated these cells with 30,000 labelled-EVs/cell for 6-15-24 hours at 37°C. The EVs were previously stained with a red-fluorescent dye which binds to lipid membranes (Vybrant™ DiI cell-labeling solution, Invitrogen), in accordance with the manufacturer’s instructions. Hoechst 33258 (Sigma-Aldrich) staining was used to visualize cellular nuclei. EVs uptake was evaluated by confocal microscopy.

### Stimulation experiments using RPTEC-derived EVs, or TOT-CM or CM and co-culture experiments

To test the role of RPTEC-derived paracrine factors in MSC differentiation, MSCs were seeded in MSCBM, at a density of 50,000 cells/well in 6-well plates (Becton Dickinson) and stimulated with 150,000 EVs/cell, 30% CM, or 30% TOT-CM for 7–14 days before RNA extraction. When using TOT-CM to stimulate MSCs, we maintained the same ratio EV:cells that we used to stimulate MSCs with EVs. For co-culture experiments, a transwell system with a 1 μm pore size permeable membrane (Becton Dickinson) was used to separate RPTECs physically from bone marrow MSCs in 6-well plates (Becton Dickinson). RPTECs were seeded into the upper insert of the transwell system in DMEM with 5% FBS, at a density of 100,000 cells/well. MSCs were seeded into the lower chamber of this co-culture system, at a density of 50,000 cells/well. MSCs cultured alone in MSCBM were used as control. Cells were maintained in co-culture conditions for 7 days before RNA extraction. Six different experiments with similar results were done.

### Trans-epithelial electric resistance

Trans-epithelial electrical resistance (TEER) was used as an indicator of epithelial differentiation and integrity (30). Control MSCs, or MSCs cultured in the presence of EVs or TOT-CM, or MSCs co-cultured with RPTECs were plated on polycarbonate membrane transwell (Falcon Corning Corp., Cambridge, MA) and allowed to reach confluency. An epithelial volt-ohm meter (EVOM, World Precision Instruments, Inc., Sarasota, FL) was used to determine the TEER. Measurement of cell-free membrane inserts were performed and subtracted from all subsequent measurement and expressed as ohm/cm^2^. All values were normalized for the area of the membrane. All experiments were done in triplicate.

### Albumin uptake

Cellular uptake of albumin was analyzed in confluent MSC monolayers using fluorescein isothiocyanate (FITC)-labeled albumin (Sigma-Aldrich), as previously described [32]. Briefly, after 14 days of co-culture with RPTECs or stimulation with EVs or TOT-CM, cell medium was refreshed and MSCs were incubated with 50 μg/ml of FITC-labeled BSA (Sigma-Aldrich) for two hours at 37°C. Fluorescence emission was measured by cytofluorimetric analysis.

### Western Blot Analysis

For protein analysis, EVs and cells were lysed at 4°C for 30 minutes in RIPA buffer (20 nM Tris-HCl, 150 nM NaCl, 1% deoxycholate, 0.1% SDS, 1% Triton X-100, pH 7.8) supplemented with PMSF, protease and phosphatase inhibitors cocktail (Sigma-Aldrich). EV samples were quantified using a proteic Bradford assay. Since the EVs protein concentration was lower than 2 mg/ml, a mixture of methanol-chloroform-water was used for the quantitative precipitation of proteins, as previously described [33,34]. Aliquots of the cell lysates containing 30 μg proteins were run on 10% acrylamide gel SDS-PAGE under reducing conditions. Proteins were transferred onto a PVDF membrane using the 7 minutes transfer program of the iBlot™ Dry Blotting System (Life Technology, Carlsbad, CA). The blots were blocked with 5% non fat milk in PBS supplemented with 0.1% Tween-20 (PBS-T). The following primary antibodies were used at 1∶200 dilution: anti-human aminopeptidase A and anti-human pan-cytokeratin (Santa Cruz Biotechnology). After incubation for 15 hours at 4°C with primary antibodies, membranes were washed with PBS-T and then incubated with for one hour at room temperature with peroxydase conjugated secondary antibodies (Santa Cruz Biotechnology). Finally, membranes were washed with PBS-T, developed with ECL detection reagents (GE healthcare, Amersham, Buckinghamshire, UK) and detected by Chemidoc XRS system (Bio-Rad).

### RNA extraction and quantitative Real Time Reverse Transcriptase Polymerase Chain Reaction

Total RNA was isolated from both EVs and cells (used in co-culture and stimulation experiments) using miRNeasy mini kit (Qiagen, Valencia, CA), according to manufacturer’s instructions, and quantified spectrophotometrically (Nanodrop ND-1000, Wilmington DE). The quality of EVs-derived RNA was evaluated by capillary electrophoresis on an Agilent 2100 Bioanalyzer (Agilent Technologies, Inc, Santa Clara, CA) using the eukaryotic total RNA 6000 Pico Kit. The presence of small RNAs was verified in both EV and cell samples using the Small RNA Kit (Agilent Tech).

The mRNA expression in MSCs co-cultured or stimulated with EVs was assessed by quantitative real-time PCR. High Capacity cDNA Reverse Transcription Kit (Applied Biosystems) and the Power SYBR® Green PCR Master Mix (Applied Biosystems) were used as previously described [26]. Negative cDNA controls (no cDNA) were cycled in parallel with each run. Quantitative real-time polymerase chain reaction (qRT-PCR) was performed using a 96-well StepOne Real-Time System (Applied Biosystems). Sequence-specific oligonucleotide primers were purchased from MWG-Biotech AG (Ebersberg, Germany, www.mwg-biotech.com) and are shown in Table 1. Fold change in mRNA expression compared to control was determined as 2^-ΔΔCt^ for all samples, using TBP as normalizer. The endogenous control in EVs was significantly different from that of their cells of origin. For this reason, miRNA comparisons between cells and EVs was not performed.

**Table 1:** Primers used in qRT-PCR to evaluate mRNAs expression.

Gene	Symbol/gene ID:	Primer sequence
Aminopeptidase A	ENPEP / 2028	Forward: 5’-GCCTTGGCAAGAGCTCAA-3’ Reverse: 5’-GCTGAAATTACTCTCTGCCATGGT-3’
Cyclin D1	CCND1 / 595	Forward: 5’-TATTGCGCTGCTACCGTTGA-3’ Reverse: 5’- CCAATAGCAGCAAACAATGTGAAA-3’
Cytokeratin 18	KRT18 / 3875	Forward: 5’-TGGCGAGGACTTTAATCTTGGT-3’ Reverse: 5’-ACCACTTTGCCATCCACTATCC-3’
Fibronectin 1	FN1 / 2335	Forward: 5’-TGAAGCTGAAGAGACTTGCTTTGA-3’ Reverse: 5’-CAGCGGTTTGCGATGGTAC-3’
FOXC2	FOXC2 / 2303	Forward: 5’-CGCCTAAGGACCTGGTGAAG-3’ Reverse: 5’-GGTAGATGCCGTTCAAGGTGAT-3’
Insulin-like growth factor 1 Receptor [35]	IGF1R / 3480	Forward: 5’-CCATTCTCATGCCTTGGTCT-3′ Reverse: 5′-TGCAAGTTCTGGTTGTCGAG-3′
Twist	TWIST1 / 7291	Forward: 5’-TCACGAGCGGCTCAGCTAC-3’ Reverse: 5’-TCTCTGGAAACAATGACATCTAGGTC-3’
Vimentin	VIM / 7431	Forward: 5’-GGAACAGCATGTCCAAATCGAT-3’ Reverse: 5’-CAGCAAACTTGGATTTGTACCATT-3’
TATA binding protein	TBP / 6908	Forward: 5’-TGTGCACAGGAGCCAAGAGT3-3’ Reverse: 5’-ATTTTCTTGCTGCCAGTCTGG-3’

### MicroRNA profiling by quantitative real-time PCR

Mature miRNA expression levels in EVs were analyzed as previously described [29,30]. The Applied Biosystems TaqMan® MicroRNA Assay Human Panel Early Access Kit (Life Technology) was used to profile 754 mature miRNAs by sequential steps of reverse transcription (Megaplex RT Pools; Life Technology) using an Applied Biosystems 7900H qRT-PCR instrument. The SDS software (version 2.3) was used to calculate raw C_T_ values, automatic baseline, and threshold. Since a C_T_ value of 35 represents single molecule template detection, C_T_ values higher than 35 were considered to be below the detection level of the assay and excluded from the analyses.

To confirm the expression of MET-related miRNAs, screened by microarray analysis, miScript Reverse Transcription Kit and miScript SYBR Green PCR Kit (both from Qiagen) were used as previously described [22]. All samples were run in triplicate and 3 ng of cDNA were used for each reaction, as described by the manufacturer’s protocol (Qiagen). MiRNAs specific primers to hsa-miR-200a, 200b, 200c, 141 and 429 are listed in Table 2 and were all used in the same reaction. The small nucleolar RNA RNU-6B was used as reference control to normalize data. A 96-well StepOne^TM^ Real Time System (Applied Biosystems) was used to perform qRT-PCR.

**Table 2:** Primers used in qRT-PCR to evaluate microRNAs expression.

microRNA	Primer sequence
hsa-miR-200a-3p	5’-TAACACTGTCTGGTAACGATGT-3’
hsa-miR-200b-3p	5’-TAATACTGCCTGGTAATGATGA-3’
hsa-miR-200c-3p	5’-TAATACTGCCGGGTAATGATGGA-3’
hsa-miR-141-3p	5’-TAACACTGTCTGGTAAAGATGG-3’
hsa-miR-429	5’-TAATACTGTCTGGTAAAACCGT-3’
hsa-RNU-6B	5’-ACGCAAATTCGTGAAGCGTT-3’

### MicroRNA target prediction and pathway analysis

The web-based application DIANA-mirPath (version 2.0) was used to perform the enrichment analysis of predicted target genes by miRNAs in biological pathways. The algorithm microT-CDS was chosen to predict EVs-derived miRNA targets, using the default microT threshold of 0.8.

DIANA-mirPath performed an enrichment analysis of multiple miRNA target genes to all known KEGG pathways, as previously described [36,37]. The statistical significance value associated with the identified biological functions was calculated by the mirPath software (http://microrna.gr/mirpath). Biological pathways showing p-value less than 0.001 were considered as significantly enriched.

### MSC transfection with miRNA mimics

MSCs were transiently transfected with miR-200a, miR-200b, miR-200c, miR-141 and miR-429 mimics (20 μM, MiScript miRNA mimics, Qiagen) using the HiPerFect Transfection Reagent (Qiagen), according to the manufacturer’s protocol. Briefly, MSCs were seeded into 6-well plates at a density of 10,000 cells/cm^2^. MiRNA mimics at a concentration of 20 nM were mixed with an appropriated volume of HiPerFect Transfection Reagent (Qiagen) and incubated at room temperature for 10 minutes. Allstars negative control siRNA (20 μM, Qiagen) was used as scrambled control. The transfection mix was added dropwise to the cells in FBS-depleted culture medium, in agreement with the manufacturer's instructions. After 24-hours of incubation, the transfection medium was removed, and cells were cultured in MSCBM for 7 days.

### Luciferase reporter assays

The pEZX-MT05 luciferase reporter vectors containing the 3′-untranslated region (UTR) sequence of human cyclin D1 (CCND1, accession number NM_053056.2) and human insulin-like growth factor 1 receptor (IGF1R, accession number NM_000875.3) inserted downstream of the luciferase reporter gene were obtained from GeneCopoeia (Rockville, MD). For luciferase reporter assays, HEK 293T cells were seeded in 24-well plates in DMEM high glucose lacking antibiotics. Luciferase-3’UTR reporter constructs (0.8 μg) or the empty expression vector (negative control) were co-transfected with miR-200a, miR-200b, miR-200c, miR-141 and miR-429 mimics (0.5 μg, MiScript miRNA mimics, Qiagen) using Lipofectamine 2000 (Invitrogen) according to the manufacturer’s instructions. After 24 hours of transfection, medium was replaced by DMEM high glucose with 10% FBS. Twenty-four hours later, firefly and Renilla luciferase activities were measured in the culture medium of the same samples using the LucPair™ miR Duo-Luciferase Assay Kit, as previously described [38].

### Statistical analysis

Statistical analysis was performed by using the t test. Statistical significance was set at *P* < 0.05.

## Results

### RPTECs induce epithelial commitment of MSCs

**Fig 1:**
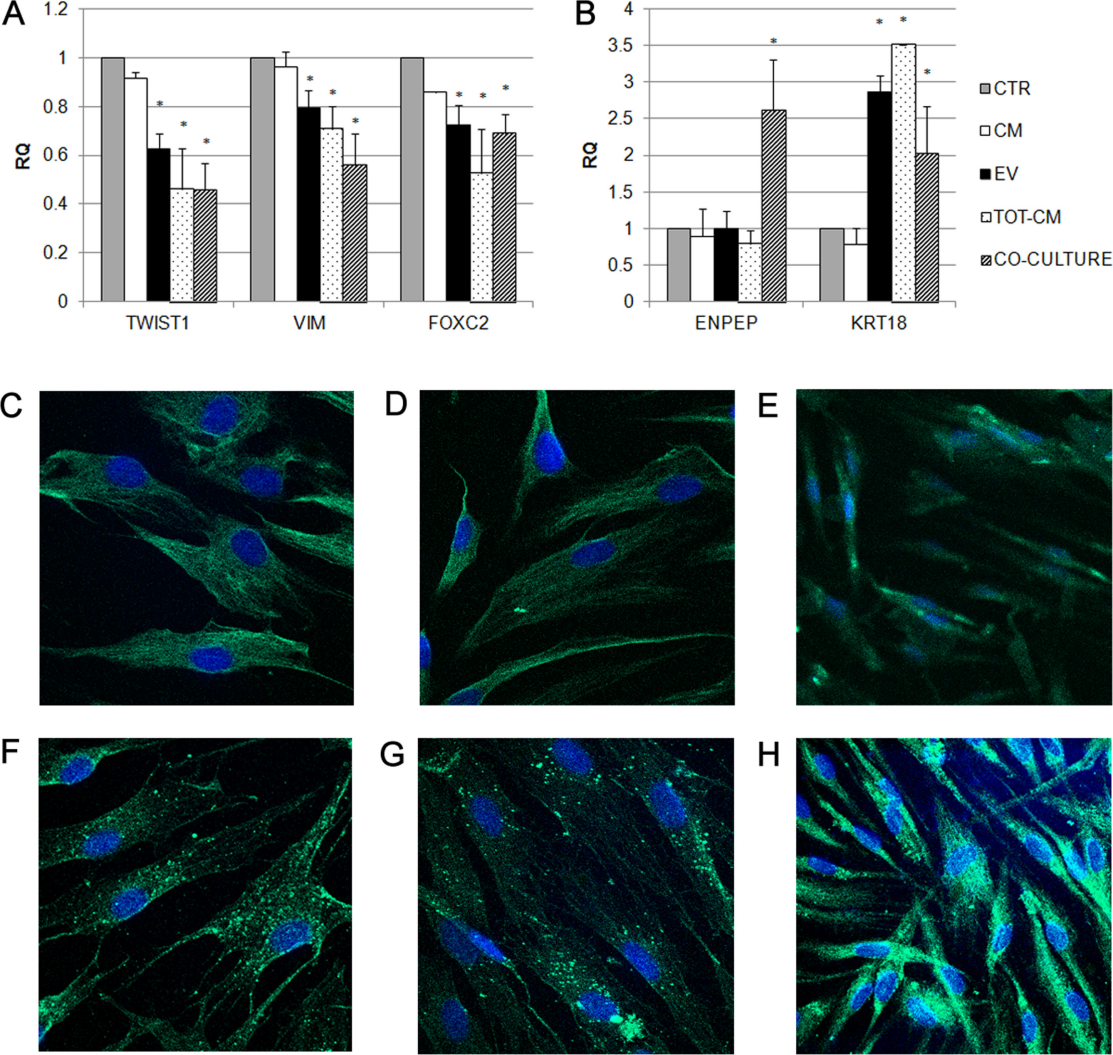
Analysis of mesenchymal and epithelial markers expression in MSCs treated for one week. (A-B) Histograms showing relative expression (RQ) in respect to control cells (CTR, grey bars) of mesenchymal markers (A) and epithelial markers (B) in MSCs, after one week of co-culture with RPTECs (striped bars), or after stimulation with EVs (black bars) or RPTEC-derived conditioned medium (TOT-CM, dotted bars) or conditioned medium deprived of EVs (CM, white bars). Results are expressed as mean of six independent experiments performed in triplicate. Data were analysed via a Student’s t test (unpaired, 2-tailed); *P<0,05 versus CTR. (C-H) Representative micrographs showing the expression of vimentin (C, D, E) and cytokeratins (F, G, H) by MSCs stimulated with EVs (C-F), TOT-CM (D-G), or co-cultured with RPTECs (E-H). Nuclei were counterstained with Hoechst dye. Six independent experiments were performed with similar results. Original magnification: X630.

To investigate the effects of RPTECs on MSCs, human bone marrow-derived MSCs and RPTECs were co-cultured in not-contact conditions. After 7 days, mRNA levels of specific mesenchymal markers Twist *(TWIST1)*, vimentin *(VIM)* and *FOXC2* were significant reduced in MSCs (Fig 1A and 1E); moreover, MSCs expressed higher levels of the epithelial-specific marker cytokeratin 18 *(KRT18)*, suggesting that MET occurred in MSCs (Fig 1B and 1H). Same results were observed when MSCs were incubated with TOT-CM (Fig 1A, 1B, 1D and 1G). To investigate the contribution of paracrine factors in epithelial differentiation of MSCs induced by RPTECs, MSCs were also incubated with CM or with purified EVs. Interestingly, after one week of incubation with CM, we did not observe any change in the expression of mesenchymal and epithelial markers in MSCs, suggesting that the contribution of soluble factors is not sufficient to induce the epithelial commitment of MSCs (Fig 1A and 1B). In MSCs stimulated with EVs, we observed a significant reduction in the expression of mesenchymal markers (Fig 1A and 1C) and an increase in the expression of the epithelial-specific marker *KRT18*, in a manner comparable with co-culture system (Fig 1B and 1F). These results indicate that EVs play a relevant role in inducing MET in MSCs.

**Fig 2:**
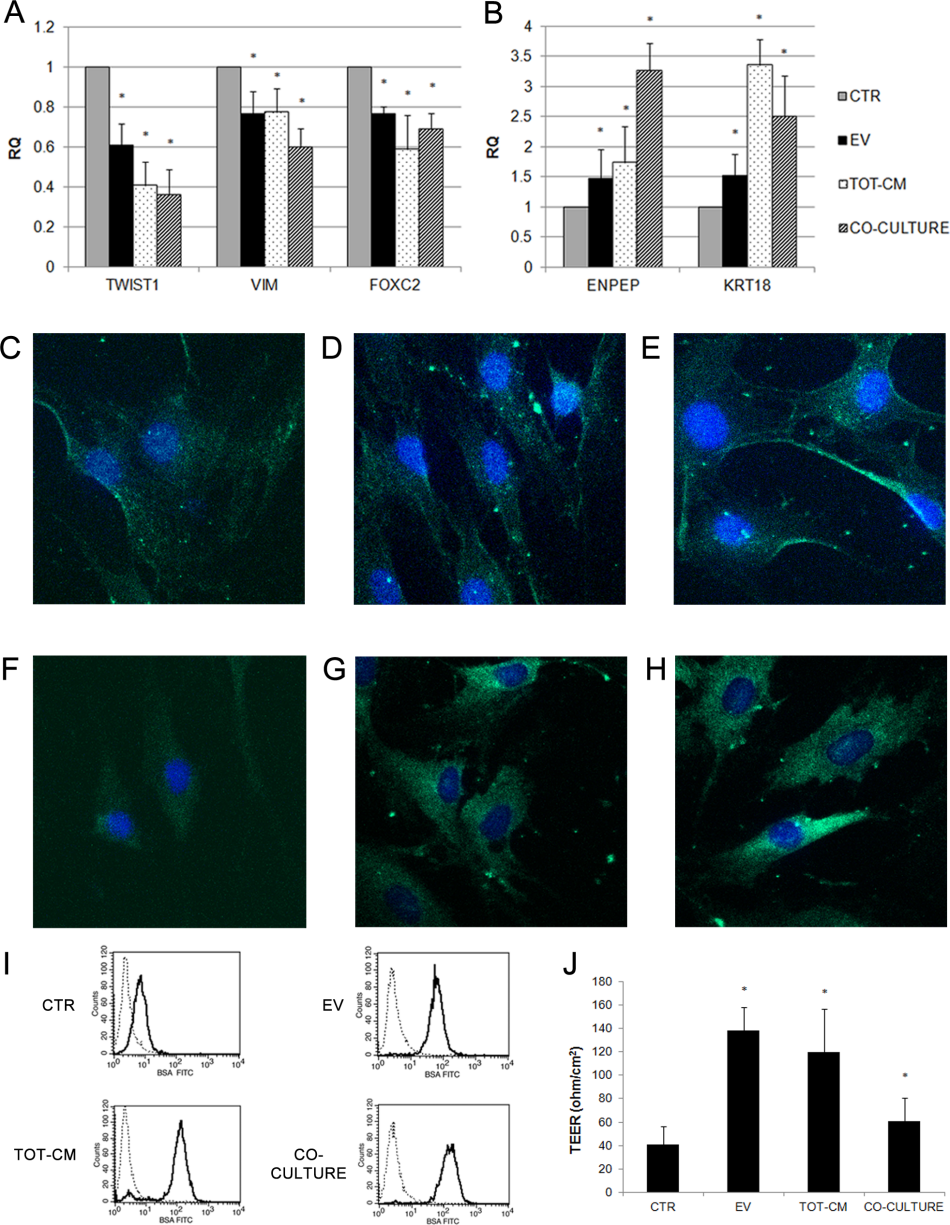
Analysis of mesenchymal and epithelial markers expression in MSCs treated for two weeks. (A-B) Histograms showing relative expression (RQ) in respect to control cells (CTR, grey bars) of mesenchymal markers (A) and epithelial markers (B) in MSCs, after two weeks of co-culture with RPTECs (striped bars), or after stimulation with EVs (black bars) or RPTEC-derived conditioned medium (TOT-CM, dotted bars). Results are expressed as mean of six independent experiments performed in triplicate. Data were analysed via a Student’s t test (unpaired, 2-tailed); *P<0.05 versus CTR. (C-H) Representative micrographs showing the expression of cytokeratin 18 (C, D, E) and aminopeptidase A (F, G, H) by MSCs stimulated with EVs (C-F), TOT-CM (D-G), or co-cultured with RPTECs (E-H). Nuclei were counterstained with Hoechst dye. Original magnification: X630. (I) Significant increased uptake of FITC-labeled albumin (BSA FITC) by MSC monolayers co-cultured with RPTECs, stimulated with EVs or with TOT-CM; *P<0.05 versus CTR. (J) Significant variation of TEER in MSC monolayers co-cultured with RPTECs, stimulated with EVs or with TOT-CM; *P<0.05 versus CTR.

Notably, the down-regulation of the expression of mesenchymal markers and the increase in cytokeratin 18 expression were also maintained after two weeks of stimulation with EVs (Fig 2A, 2B and 2C), TOT-CM (Fig 2A, 2B and 2D) and of co-culture with RPTECs (Fig 2A, 2B and 2E). A slight but significant increase in expression of the renal tubular-specific marker aminopeptidase A *(ENPEP)* was also observed in MSCs stimulated with EVs (Fig 2B and 2F), TOT-CM (Fig 2B and 2G). The early expression of *ENPEP* in MSCs co-cultured with RPTECs (Fig 1B) was also maintained after 14 days (Fig 2B and 2H), suggesting that the renal tubular commitment of MSCs was facilitated by a bidirectional exchange of paracrine factors between the two cell populations.

Human proximal tubular epithelial cells can effectively reabsorb albumin by receptor-mediated endocytosis [39]. To verify whether this tubular epithelial-specific function was induced by EVs in MSCs, we quantified fluorescently-labeled albumin uptake using cytofluorimetric analysis. MSCs stimulated with EVs, TOT-CM and co-cultured with RPTECs successfully incorporated FITC-labeled albumin (Fig 2I). Moreover, evaluation of TEER as an indicator of epithelial differentiation and integrity showed that EVs and TOT-CM induced a significant increase of TEER in MSCs (Fig 2J). Taken together, these results confirm that EVs can induce the epithelial commitment of MSCs.

### Characterization of EVs and incorporation into MSCs

**Fig 3:**
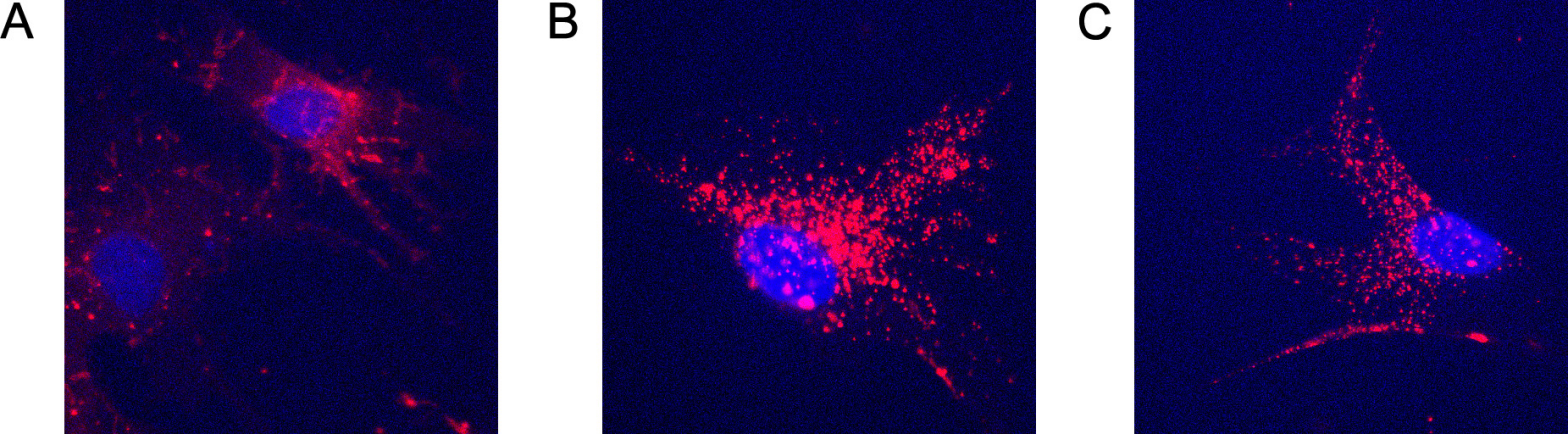
Incorporation of EVs in MSCs. Representative micrographs of internalization by MSCs of EVs stained with Vybrant Dil (red) at 37°C for 6 hours (A), 15 hours (B), 24 hours (C) observed by Confocal microscopy. Z-stack was performed to verify the effective internalization of EVs. Nuclei were counterstained with Hoechst dye. Three experiments were performed with similar results. Original magnification: X630.

To better clarify the mechanisms underlying the epithelial commitment induced by RPTECs on MSCs, we evaluated the capacity of EVs to be incorporated into MSCs and characterized EVs for the presence of specific proteins and mRNAs. Starting from 6 hours of incubation, labeled EVs were internalized by MSCs, with a progressive compartmentalization of EVs inside the cell at 15 and 24 hours of incubation (Fig 3).

**Fig 4:**
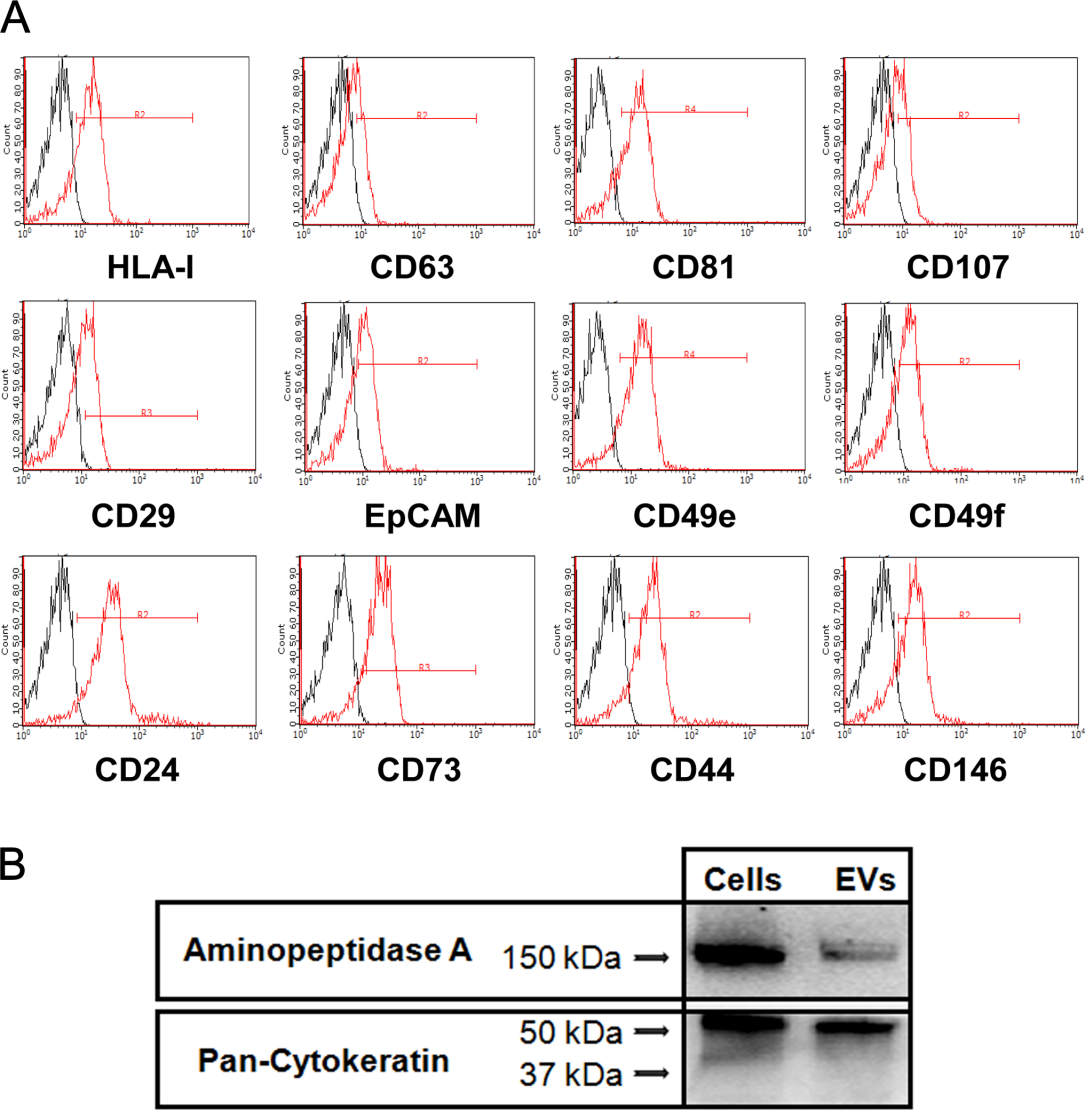
Characterization of EVs protein expression. (A) Characterization of EVs by cytofluorimetric analysis. Representative FACS analyses of EVs showing the expression of CD24, CD29, CD44, alpha-5 integrin (CD49e), alpha-6 integrin (CD49f), CD73, CD146, EpCAM, and HLA-class I surface molecules and classic exosomal markers CD63, CD81, CD107 (thick lines). Dotted lines indicate the isotypic controls. Three different EV preparations were analyzed with similar results. (B) Representative western blot analysis on RPTEC cells and EVs. Three different experiments were performed with similar results.

Cytofluorimetric analysis showed that EVs carried several surface adhesion molecules also expressed on the plasma membrane of RPTECs, such as CD24, CD29, CD44, CD73, CD146, alpha-5 integrin (CD49e), alpha-6 integrin (CD49f), HLA1 and EpCAM. EVs also expressed classical exosomal markers CD63, CD81 and CD107a (LAMP1) (Fig 4A). Moreover, we found that EVs shuttled both epithelial-specific proteins and mRNA, such as cytokeratins, and the tubular-specific marker ENPEP (Fig 4B and Table 3).

**Table 3:** Analysis of epithelial-specific mRNAs expression by RPTEC cells and EVs. Results are expressed as mean ± SD of three independent experiments.

mRNA	Cells		EVs	
	C_T_ Mean	C_T_ SD	C_T_	C_T_ SD
**ENPEP**	23.16	0.19	31.97	0.72
**KRT7**	23.05	0.69	30.43	1.48
**KRT18**	22.10	0.36	28.27	0.42
**KRT19**	20.48	1.39	27.32	0.69

**Fig 5:**
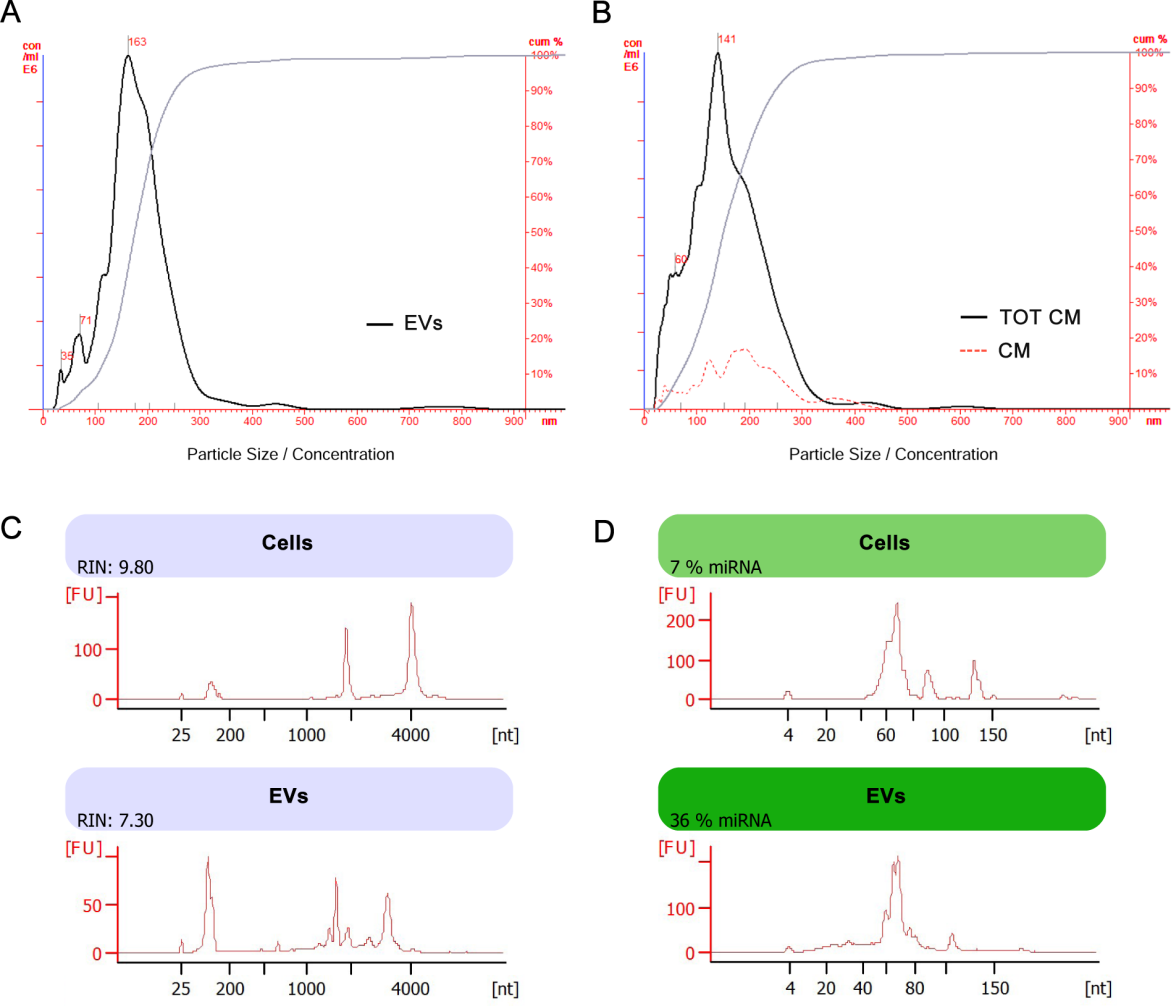
Characterization of EVs size and RNA content. (A-B) Representative EV size analysis by direct measurement with NTA, in EVs (A) and in RPTEC-derived conditioned medium with (TOT-CM) or without EVs (CM) (B) by direct measurement with NTA, showing the difference of EV concentration before and after EV depletion. (C) Representative Bioanalyzer profiles, showing the size distribution of total RNA extracted from EVs and cells. The first peak (left side of each panel) represents an internal standard. Whereas the two peaks of ribosomal RNA 18S and 28S were detectable in cells, they were barely detectable in the corresponding EVs. Unlike the cells of origin, EVs exhibited a relevant peak of small RNAs. RNA Integrity Number (RIN) is shown for both cells and EVs. (D) Representative bioanalyzer profile of small RNAs performed on EVs, showing an enrichment of the miRNA fraction (range: 33–36%) in respect to the cells of origin (range: 4–7%). Three different samples tested in triplicate were analyzed for both EVs and cells with similar results.

Nanosight analysis revealed that the average size of EVs was 185 nm with a mode of 163 nm and a standard deviation of 84 nm (Fig 5A). EVs presence was also confirmed in TOT-CM, with an average size of 162 nm, a mode of 141 nm and a standard deviation of 84 nm (Fig 5B). After RPTEC supernatant ultracentrifugation, EVs concentration was highly reduced in CM (Fig 5B).

### MicroRNA profiling in EVs and comparative pathway analyses

We verified the quality of EVs-derived RNA and the presence of small RNAs, with a bioanalyzer profile on total RNA from EVs and their cell of origin. EVs contained a broad range of RNA sizes, with a RNA Integrity Number (RIN) of 7.30 out of 10 (Fig 5C). A more comprehensive analysis using a bioanalyzer kit specific for small RNAs showed a relevant peak characteristic of small RNA classes inside EVs, with an enrichment of miRNAs around 36% (Fig 5D). RNA extracted from EVs was profiled for 754 known human mature miRNAs. We found 237 miRNAs expressed (S1 Table). Among them, we found a subset of five miRNAs (miR-200a, miR-200b, miR-200c, miR-141 and miR-429) that belong to miR-200 family, known to be involved in EMT [40,41].

In order to examine which biological pathways may be involved in the regulation of the epithelial commitment of target cells, we applied the software DIANA MirPath on the 65 most expressed miRNAs derived from EVs. We performed an enrichment analysis of predictive miRNA target genes included in KEGG database and we found that 63 KEGG biological processes were significantly enriched (p<0.001, FDR corrected) (S2 Table). Twenty miRNAs found in EVs revealed a strong association to several biological pathways that are known to modulate EMT (Table 4).

**Table 4:** MiRNAs shuttled by EVs and associated to EMT-related KEGG pathways. Results are expressed as mean ± SD of three independent experiments.

miRNA	C_T_ Mean	C_T_ SD
hsa-miR-1305	18.20	0.66
hsa-miR-17-5p	20.67	0.74
hsa-miR-19b-3p	20.68	1.08
hsa-miR-106a-5p	20.77	0.78
hsa-miR-29a-3p	20.96	0.47
hsa-miR-302a-3p	21.24	0.29
hsa-miR-16-5p	21.45	0.94
hsa-miR-320a	21.79	0.24
hsa-miR-186-5p	21.82	0.27
hsa-miR-374b-5p	21.98	0.23
hsa-miR-200b-3p	22.87	0.21
hsa-let-7e-5p	23.31	0.24
hsa-miR-374a-5p	23.88	0.07
hsa-miR-20a-5p	23.90	1.15
hsa-miR-30a-5p	24.17	0.85
hsa-miR-520a-3p	24.57	0.75
hsa-miR-19a-3p	24.60	1.33
hsa-miR-429	24.63	0.32
hsa-let-7b-5p	24.78	0.22
hsa-let-7g-5p	24.88	0.29

**Fig 6:**
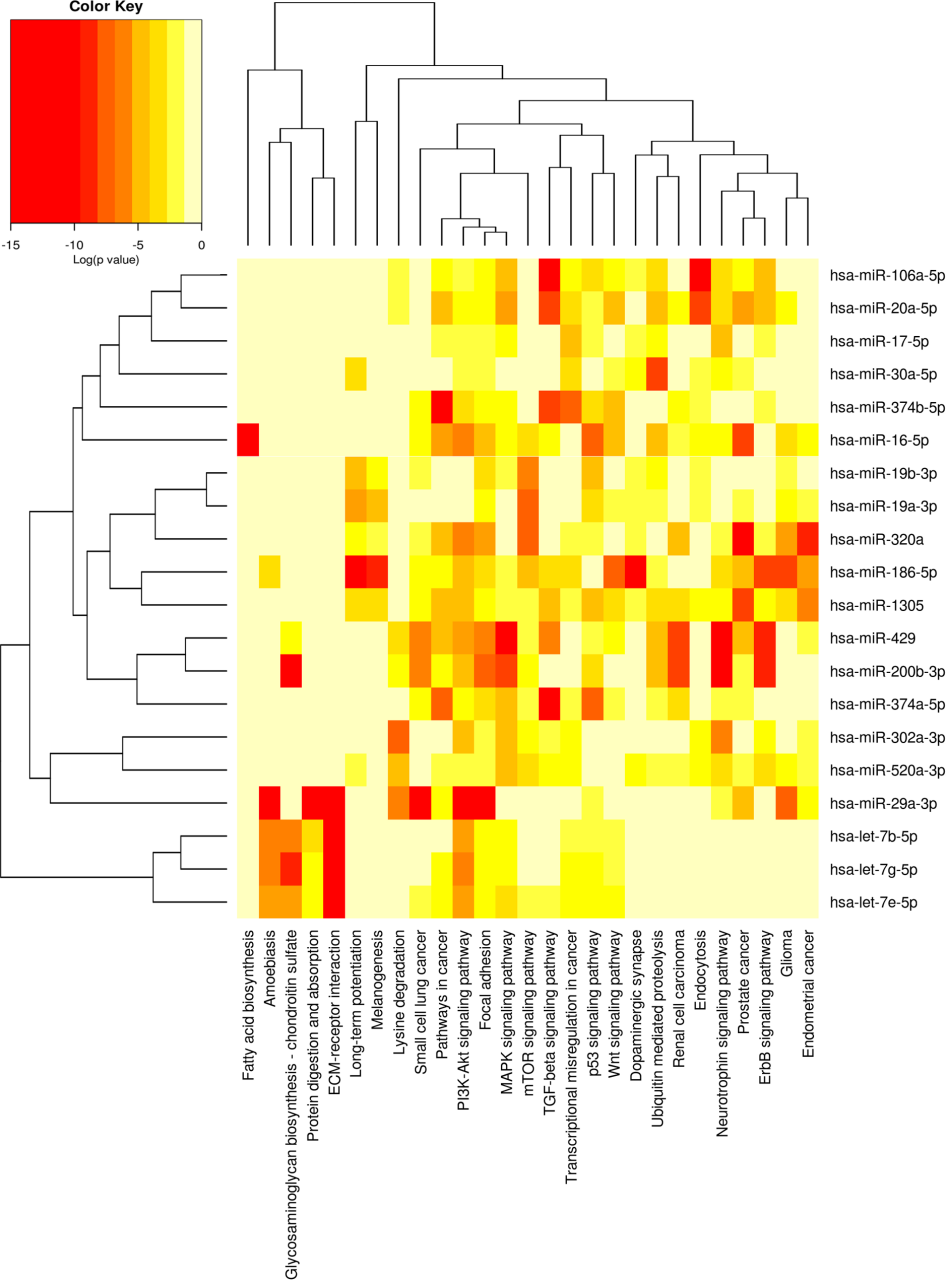
Heat map showing miRNAs versus pathways. Using the option *union of pathways*, DIANA MirPath provided a graphical overview of pathways enriched by the 20 selected EVs-derived miRNAs. Darker colors represent lower significance values. MET-related pathways (highlighted by the circle line) are targeted by the selected miRNAs with a very low p-value (p<10^−7^, FDR corrected). The attached dendrograms illustrate hierarchical clustering results for miRNAs and pathways. MiRNAs that show similar pathway targeting patterns were clustered together. An analogous clustering was also performed for the biological pathways.

Our analysis showed that the predicted target genes of these miRNAs are principally involved into the following EMT and MET-associated pathways: ECM-receptor interaction, Wnt pathway, gap junction, transcriptional misregulation in cancer, TGF-beta pathway, pathways in cancer, MAPK signaling pathway, p53 signaling pathway, focal adhesion, PI3K-Akt signaling pathway, regulation of actin cytoskeleton (Table 5) [11,42–45]. Fig 6 provides a graphical representation of the most significantly enriched pathways modulated by the 20 miRNAs that we selected from our analysis.

**Table 5:** Biologic pathways enriched by 20 selected miRNAs found in EVs, that contain EMT-related genes.

KEGG pathway	p-value	# genes	#miRNAs
ECM-receptor interaction	< 1 E-16	20	4
Wnt signaling pathway	< 1 E-16	66	5
Gap junction	< 1 E-16	30	5
Transcriptional misregulation in cancer	< 1 E-16	60	5
TGF-beta signaling pathway	< 1 E-16	41	7
Pathways in cancer	< 1 E-16	126	7
MAPK signaling pathway	< 1 E-16	84	8
p53 signaling pathway	< 1 E-16	33	8
Focal adhesion	< 1 E-16	94	8
PI3K-Akt signaling pathway	< 1 E-16	159	12
Regulation of actin cytoskeleton	< 1 E-16	20	1
HIF-1 signaling pathway	5.076362 E-08	17	1
Hedgehog signaling pathway	1.950418 E-05	15	2

**Fig 7:**
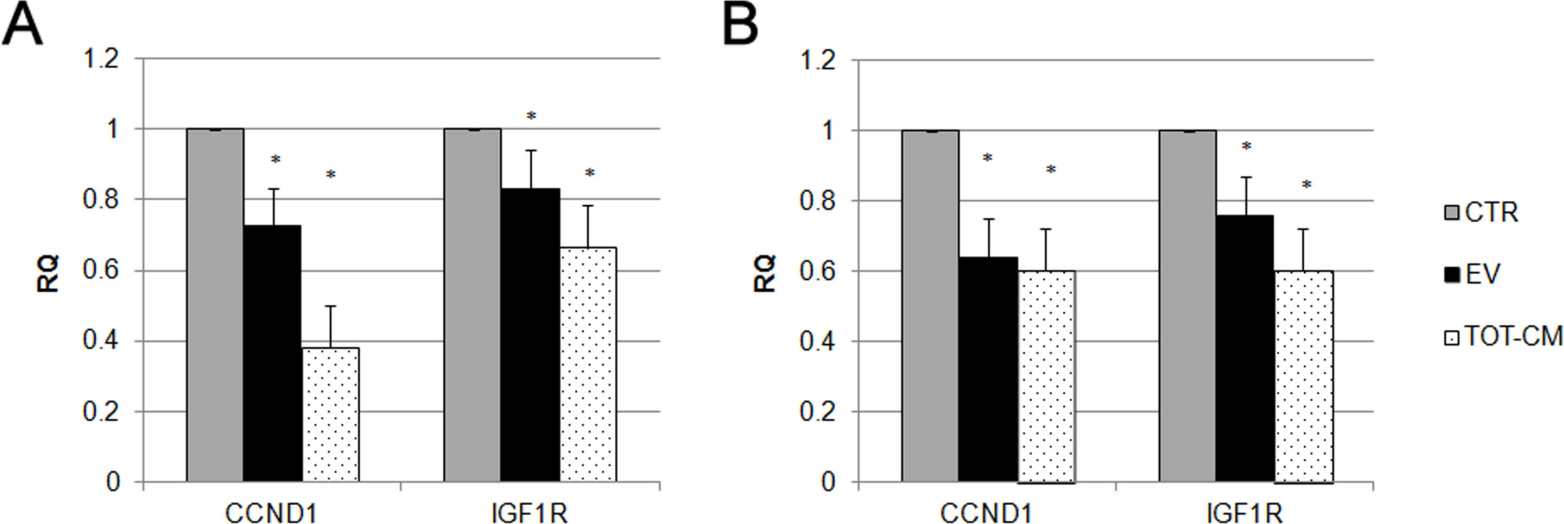
Analysis of miRNA predictive target genes in MSCs stimulated with EVs. Histograms showing relative expression (RQ) of miRNA predictive target genes in MSCs, after one (A) and two weeks (B) of stimulation with EVs (black bars), or RPTEC-derived conditioned medium (TOT-CM, dotted bars), in respect to control cells (CTR, grey bars). Results are expressed as mean of six independent experiments performed in triplicate. Data were analyzed via a Student’s t test (unpaired, 2-tailed); *P<0.05 versus CTR.

We selected two miRNA targets from our predictive analysis, *CCND1* and *IGF-1R*, and examined their expression in MSCs after one week of incubation with EVs or TOT-CM. In both conditions, we observed a reduction in the expression of *CCND1* and *IGF-1R* in MSCs, that could be attributed to miRNA-mediated inhibitory effects (Fig 7). These data suggest that EVs may prompt an epithelial commitment in target cells, by the delivery of specific miRNAs involved in MET-related pathways.

### Role of the miR-200 family in the epithelial commitment of MSCs

**Fig 8:**
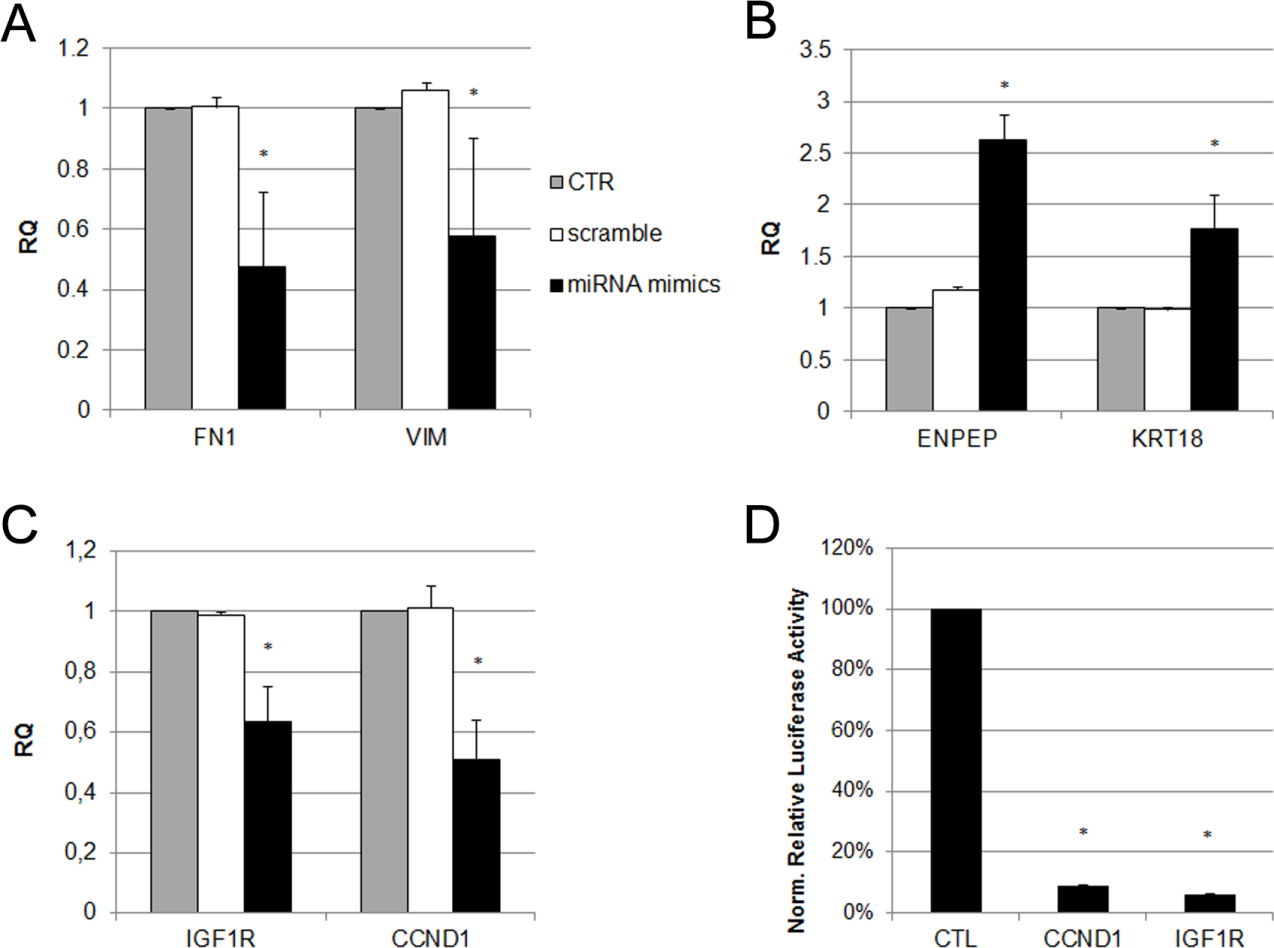
Analysis of mesenchymal and epithelial markers expression in MSCs transfected with miR-200 mimics. Histograms showing relative expression (RQ) of mesenchymal markers (A), epithelial markers (B) and miRNA predictive target genes (C) in MSCs, after one week of transfection with miR-200 mimics (black bars), or with a control miRNA (scramble, white bars), in respect to control cells (CTR, grey bars). Results are expressed as mean of three independent experiments performed in triplicate. Data were analysed via a Student’s t test (unpaired, 2-tailed); *P<0.05 versus CTR. (D) Luciferase expression in HEK 293T cells following co-transfection of *CCND1* or *IGF1R* 3’UTR reporter constructs and miR-200 family mimics for 24 hours. Firefly luciferase expression is normalized to Renilla luciferase for each gene-specific 3’UTR and presented as a percentage of the luciferase activity variation in respect to the same cells co-transfected with a negative control vector (CTR). Values are expressed as mean of three independent experiments; *P<0.05 versus CTR.

We employed qRT-PCR to validate the expression levels of the five miRNAs belonging to miR-200 family, selected from the miRNA array. We showed that all these five miRNAs were strongly expressed in EVs and we selected them to study their possible role in MET induction in MSCs. A mixture of mimics was used to increase the expression of miR-200a, miR-200b, miR-200c, miR-141 and miR-429 in MSCs. After 1 week of transfection, MSCs showed lower levels of MET markers *VIM* and fibronectin 1 (*FN1)* (Fig 8A), and higher levels of epithelial marker *KRT18* and tubular marker *ENPEP* (Fig 8B). Moreover, MSCs transfection with miR-200 mimics reduced the expression of two miRNA targets from our predictive analysis, *CCND1* and *IGF-1R* (Fig 8C).

To assess whether miR-200 family can effectively repress translation through binding to *CCND1* and *IGF1R* 3’UTR, we performed luciferase reporter assay. MiR-200 family mimics were co-transfected in HEK 293T cells with the luciferase reporter construct pEZX-MT05 containing the human *CCND1* or *IGF1R* 3’UTR immediately following the luciferase coding sequence. As a result, miR-200 family induced a 90% reduction of luciferase activity in transfected cells.

Taken together, these results suggest that the miR-200 family carried by EVs can induce MET in MSCs by binding to the 3’UTR sequences of predicted target genes, like CCND1 and IGF1R (Fig 8D).

## Discussion

The mechanism of cell communication mediated by EVs allows exchange of proteins and genetic information between cells, as EVs may transfer mRNAs, long non coding RNAs (lncRNAs) and miRNAs to target cells [18,20,46–48]. The epithelial commitment of bone marrow-derived cells induced by EVs derived from differentiated tissues has been demonstrated by Quesenberry’s group [49]. They showed that EV-mediated transfer of proteins and genetic information from lung injured cells to bone marrow-derived cells may reprogram marrow cell phenotype to acquire lung epithelial-specific markers [19,20]. Moreover, EVs derived from prostate or lung cancer cells and EVs derived from lung injured cells were also shown to trigger stable epigenetic changes in marrow cells, activating the expression of prostate- or lung-specific genes [50,51], following internalization of EVs into target cells [52]. Taken together, these results indicate that EVs collected from differentiated injured tissues can effectively induce epigenetic modifications of marrow cells, that acquire phenotypic characteristics of the tissue of origin.

Previous studies have shown that tubular epithelial cells and their conditioned medium may induce an epithelial transition of MSCs [1–4]. In the present study, we have demonstrated that EVs released by RPTECs are the main mediators of the epithelial differentiation of bone marrow-derived MSCs. In fact, we noticed that RPTEC-derived conditioned medium depleted of EVs was no longer able to induce MET in MSCs. In particular, after one week of incubation of MSCs with EVs, we observed the decreased expression of TWIST1, FOXC2, known to repress epithelial genes expression [53,54], and of vimentin, a protein involved in cellular adhesion and migration [55,56]. Moreover, after two weeks of MSC stimulation with EVs, we observed the increased expression of epithelial marker cytokeratin 18, which contributes to maintenance of epithelial cell polarity and plays an important role in cellular adhesion and cell-to-cell interactions [57–59], and a slight increase in the expression of aminopeptidase A. This zinc-dependent metalloproteinase is typically expressed in the brush border membrane of renal proximal tubule cells and it is known to be involved into the removal of N-terminal acid aminoacidic remainings from different peptides, for example angiotensin II [60,61]. Taken together, these results indicate an epithelial differentiation of MSCs induced by EVs.

To understand the mechanism of epithelial commitment of MSCs induced by EVs, we characterized EV protein and RNA content. We found that EVs contained epithelial-specific proteins and transcripts that after delivering to target cells may be responsible for the early epithelial commitment of MSCs. The persistence of *KRT18* expression in MSCs after 14 days of stimulation with EVs suggests that epigenetic changes contributed to maintain MSCs epithelial commitment. The long-term modification in MSC transcriptome was probably due to epigenetic changes that follow the transfer of non-coding RNAs, such as miRNAs [62]. Recently, the EVs-mediated transfer of miRNAs has been observed between differentiated normal cells [21,63], between MSCs and differentiated cells [64–66], between MSCs and cancer cells [67–69] and inside the tumor microenvironment [70–72].

To investigate the miRNA content of EVs, we profiled their RNA for 754 known human mature miRNAs and we identified 20 miRNAs with a strong association to EMT pathways. In particular, we detected the expression of some miRNAs belonging to miR-200 family. Recent studies on tumor cells [73–76] have demonstrated that miR-200 family miRNAs (miR-200a, miR-200b, miR-200c, miR-141, miR-429) are involved in EMT. In fact, mature miR-200 family miRNAs promote E-cadherin expression with the acquisition of an epithelial cell phenotype via post-transcriptional repression of zinc finger E-box binding transcription factor 1 and 2 (*ZEB1* and *ZEB2*) [77]. During EMT, *ZEB1* and *ZEB2* over-expression leads to transcriptional repression of E-cadherin, promoting a mesenchymal cell phenotype. This process may be reversible, since an increase in miR-200 family miRNAs expression initiates MET, restoring E-cadherin expression and promoting an epithelial phenotype [77].

Since miR-200 family miRNAs are shuttled by EVs, we investigated the effects of these miRNAs in MSCs and their possible contribution to MET. After one week of transfection of bone marrow derived-MSCs with 5 synthetic miR-200 mimics, we observed a reduction in the expression of *VIM* and *FN1*, which is another mesenchymal marker involved in cell adhesion, cell migration, cell growth and cell differentiation [78]. Moreover, we observed an increased expression of *ENPEP*, indicating that the miR-200 miRNAs can induce the epithelial commitment of MSCs. These data suggest that EVs may contribute to the epithelial commitment of MSCs by transferring a small subset of miRNAs that belong to the miR-200 family.

Furthermore, in order to validate the results of our predictive analysis on EV-derived miRNAs, we randomly selected cyclin D1 *(CCND1)* and insulin growth factor-1 receptor *(IGF1R)*, from all predictive miRNA target genes.

*CCND1* encodes a regulatory subunit of the cyclin-dependent kinase 4 (CDK4) and of the cyclin-dependent kinase 6 (CDK6), whose activity is required for cell cycle G1/S transition [79]. CCND1 is involved in cell proliferation, survival, invasion, metastasis formation and its over-expression is linked to tumorigenesis [80,81]. Recent studies have demonstrated that CCND1 activation can induce EMT in tumor cells, such as ovarian cancer [82], breast cancer [83], esophageal cancer [84] and epidermoid carcinoma cells [85]. In particular, Su *et al*. [84] have shown that the inhibition of CCND1 down-regulates the expression of other mesenchymal markers and reverses EMT in esophageal cancer cells.

IGF1R is a trans-membrane receptor tyrosine kinase which binds to its ligand IGF1, leading to activation of the PI3K-Akt pathway and the Erk-MAPK pathway. Previous studies have shown that IGF1R activation can induce EMT in prostate cancer [86], breast cancer [87,88], mammary epithelial cells [89] and lung cancer cells [34,90]. Nurwidya *et al*. [35] found that inhibition of IGF1R reversed hypoxia-induced EMT. Zhou *et al*. [90] reported that IGF1R activation induces EMT in lung cancer cells by up-regulating the expression of Snail and promoting beta-catenin translocation from the cell membrane into the nucleus, which, in turn, down-regulates E-cadherin expression.

We evaluated the expression of *CCND1* and *IGF1R* in MSCs after incubation with EVs or TOT-CM and after cell transfection with miR-200 mimics. We showed that both EVs and miR-200 family miRNAs can effectively reduce *CCND1* and *IGF1R* expression in MSCs. These results confirmed that *CCND1* and *IGF1R* down-regulation is indicative of MET. Luciferase reporter assay has proved that both *CCND1* and *IGF1R* are target genes of EV-delivered miRNAs, thus validating the results of our miRNA predictive analysis.

In conclusion, EVs released from tubular epithelial cells may modify the phenotype of MSCs by inducing an epithelial commitment that may contribute to the regenerative potential of MSCs. Whether this phenomenon occurs *in vivo* remains to be defined.

## Supporting Information

S1 Fig Characterization of human bone marrow-derived MSCs. A-B) Representative micrographs showing the expression of vimentin (A) and cytokeratins (B) by MSCs. Nuclei were counterstained with Hoechst dye. Three independent experiments were performed with similar results. Original magnification: X630. C) Representative FACS analyses of MSCs showing the expression of CD29, CD44, CD73, CD90 and CD105 surface molecules (blue area). The expression of EpCAM was not detected. Green lines indicate the isotypic controls. Six different MSCs preparations were analyzed with similar results.
(TIF)

S2 Fig Characterization of human renal tubular cells (RPTECs). A-B) Representative micrographs showing the expression of vimentin (A) and cytokeratins (B) by MSCs. Nuclei were counterstained with Hoechst dye. Three independent experiments were performed with similar results. Original magnification: X630. C) Representative FACS analyses of RPTECs showing the expression of CD24, CD29, CD44, alpha-5 integrin (CD49e), alpha-6 integrin (CD49f), CD73, CD146, EpCAM, and HLA-class I surface molecules (blue area). The expression of VEGF receptor II (KDR), CD45 and CD105 was not detected. Dotted lines indicate the isotypic controls. Six different RPTECs preparations were analyzed with similar results.
(TIF)

S1 Table List of 237 miRNAs found in EVs. Results are expressed as mean ± SD of three independent experiments.
(DOCX)

S2 Table List of KEGG biological pathways significantly enriched (p<0.001, FDR corrected) by miRNAs found in EVs. (DOCX)

## Abbreviations

CM: Conditioned Medium deprived of extracellular vesicles
EMT: Epithelial-Mesenchymal Transition
EVs: Extracellular Vesicles derived from RPTECs
MET: Mesenchymal-Epithelial Transition
MiRNA: MicroRNA
MSCs: Mesenchymal Stromal Cells
qRT-PCR: quantitative Real Time Reverse Transcriptase Polymerase Chain Reaction
RPTECs: Renal Proximal Tubular Epithelial Cells
TEER: Trans-Epithelial Electric Resistance
TOT-CM: Total Conditioned Medium
