# Supplementary figures and images for: Mesenchymal Stromal Cells Epithelial Transition Induced by Renal Tubular Cells-Derived Extracellular Vesicles
Source: PLoS One. 2016 Jul 13;11(7):e0159163. doi: 10.1371/journal.pone.0159163 (PMC4943710; doi:10.1371/journal.pone.0159163)

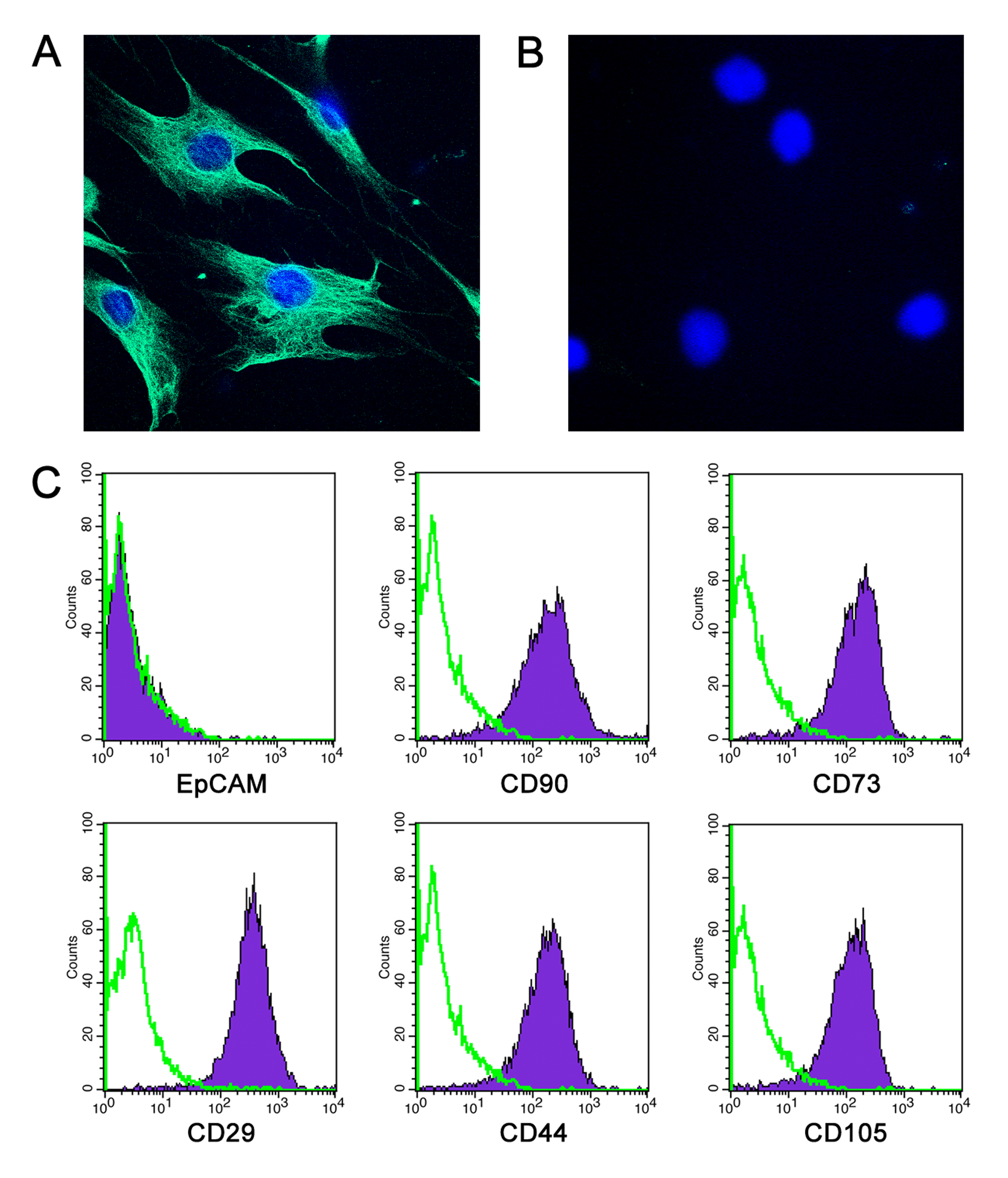

Supplement: S1 Fig — A-B) Representative micrographs showing the expression of vimentin (A) and cytokeratins (B) by MSCs. Nuclei were counterstained with Hoechst dye. Three independent experiments were performed with similar results. Original magnification: X630. C) Representative FACS analyses of MSCs showing the expression of CD29, CD44, CD73, CD90 and CD105 surface molecules (blue area). The expression of EpCAM was not detected. Green lines indicate the isotypic controls. Six different MSCs preparations were analyzed with similar results. (TIF) [file pone.0159163.s001.tif]

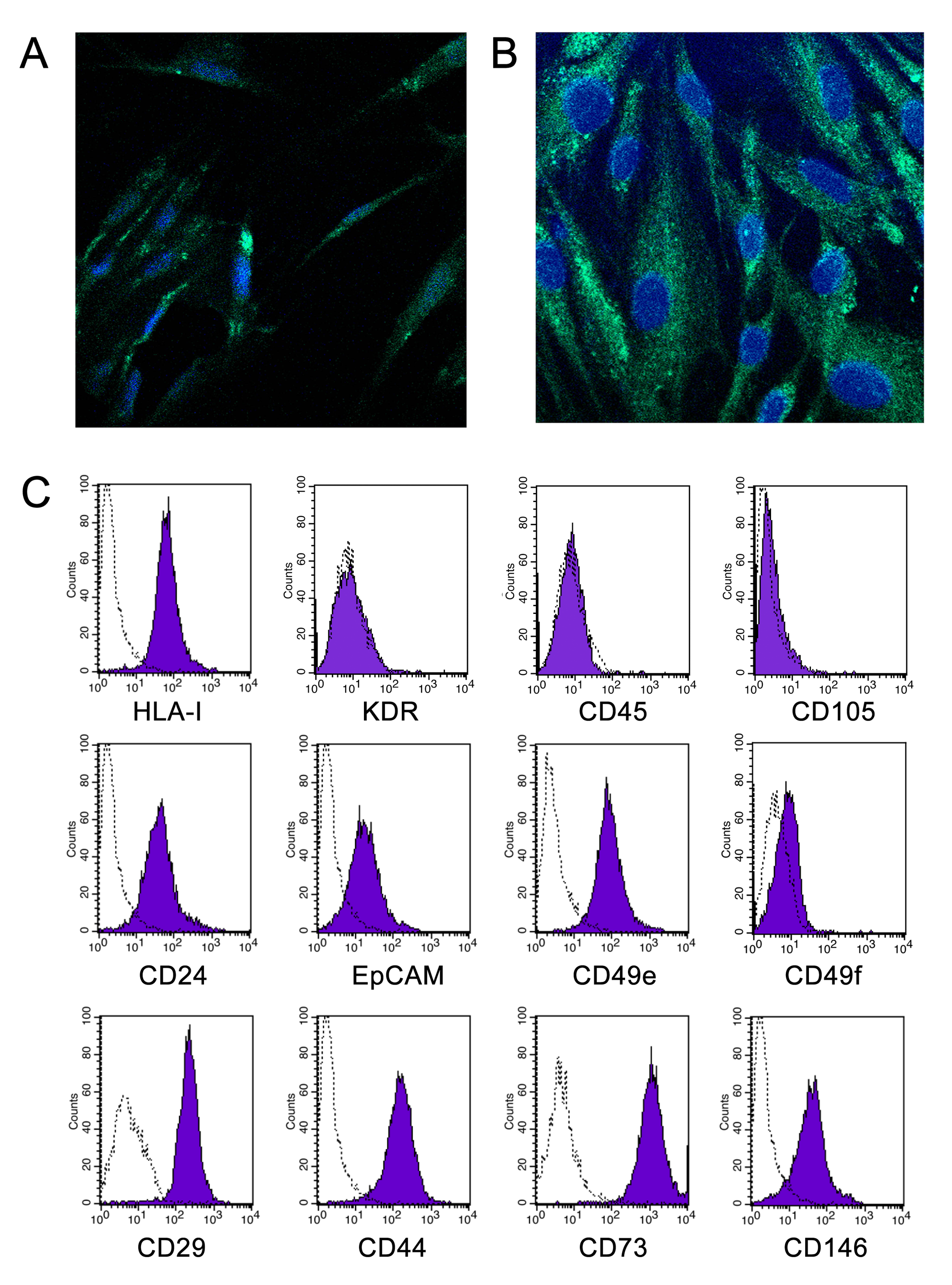

Supplement: S2 Fig — A-B) Representative micrographs showing the expression of vimentin (A) and cytokeratins (B) by MSCs. Nuclei were counterstained with Hoechst dye. Three independent experiments were performed with similar results. Original magnification: X630. C) Representative FACS analyses of RPTECs showing the expression of CD24, CD29, CD44, alpha-5 integrin (CD49e), alpha-6 integrin (CD49f), CD73, CD146, EpCAM, and HLA-class I surface molecules (blue area). The expression of VEGF receptor II (KDR), CD45 and CD105 was not detected. Dotted lines indicate the isotypic controls. Six different RPTECs preparations were analyzed with similar results. (TIF) [file pone.0159163.s002.tif]
