# Supplementary material for: Mesenchymal Stromal Cells Epithelial Transition Induced by Renal Tubular Cells-Derived Extracellular Vesicles
Source: PLoS One. 2016 Jul 13;11(7):e0159163. doi: 10.1371/journal.pone.0159163 (PMC4943710; doi:10.1371/journal.pone.0159163)
Supplement: S1 Table — Results are expressed as mean ± SD of three independent experiments. (DOCX) [file pone.0159163.s003.docx]

| **miRNA** | **C_T_ Mean** | **C_T_ SD** |
| --- | --- | --- |
| U6 snRNA | 18.76 | 1.29 |
| RNU44-001094 | 28.70 | 0.74 |
| RNU48-001006 | 22.83 | 0.80 |
| hsa-let-7a-5p | 29.97 | 1.65 |
| hsa-let-7b-5p | 24.78 | 0.22 |
| hsa-let-7c | 28.90 | 0.20 |
| hsa-let-7d-5p | 26.38 | 0.12 |
| hsa-let-7e-5p | 23.31 | 0.24 |
| hsa-let-7f-5p | 29.54 | 0.17 |
| hsa-let-7g-5p | 24.88 | 0.29 |
| hsa-miR-7-1-3p | 29.50 | 0.15 |
| hsa-miR-10a-5p | 24.51 | 0.43 |
| hsa-miR-10b-3p | 26.41 | 0.88 |
| hsa-miR-10b-5p | 24.68 | 1.18 |
| hsa-miR-15b-5p | 27.13 | 0.78 |
| hsa-miR-16-5p | 21.45 | 0.94 |
| hsa-miR-17-5p | 20.67 | 0.74 |
| hsa-miR-18a-3p | 33.05 | 1.55 |
| hsa-miR-18a-5p | 27.69 | 1.18 |
| hsa-miR-19a-3p | 24.60 | 1.33 |
| hsa-miR-19b-3p | 20.68 | 1.08 |
| hsa-miR-19b-1-5p | 33.25 | 0.48 |
| hsa-miR-20a-3p | 33.51 | 0.21 |
| hsa-miR-20a-5p | 23.90 | 1.15 |
| hsa-miR-20b-5p | 24.84 | 0.95 |
| hsa-miR-21-5p | 21.02 | 0.02 |
| hsa-miR-22-3p | 29.92 | 1.43 |
| hsa-miR-22-5p | 33.41 | 0.92 |
| hsa-miR-24-3p | 19.46 | 0.38 |
| hsa-miR-25-3p | 27.12 | 0.18 |
| hsa-miR-26a-5p | 23.36 | 0.19 |
| hsa-miR-26b-3p | 32.88 | 0.78 |
| hsa-miR-26b-5p | 25.86 | 0.17 |
| hsa-miR-27a-3p | 26.20 | 0.36 |
| hsa-miR-27a-5p | 31.63 | 0.19 |
| hsa-miR-27b-3p | 27.08 | 0.10 |
| hsa-miR-28-3p | 23.70 | 0.33 |
| hsa-miR-28-5p | 25.81 | 0.60 |
| hsa-miR-29a-3p | 20.96 | 0.47 |
| hsa-miR-29b-3p | 31.19 | 1.73 |
| hsa-miR-29c-3p | 27.32 | 0.59 |
| hsa-miR-30a-3p | 21.20 | 0.45 |
| hsa-miR-30a-5p | 24.17 | 0.85 |
| hsa-miR-30b-5p | 20.57 | 0.17 |
| hsa-miR-30c-5p | 19.94 | 0.07 |
| hsa-miR-30d-5p | 28.45 | 1.20 |
| hsa-miR-30e-3p | 21.52 | 0.56 |
| hsa-miR-31-3p | 25.35 | 1.44 |
| hsa-miR-31-5p | 20.36 | 0.32 |
| hsa-miR-32-5p | 31.90 | 1.50 |
| hsa-miR-34a-3p | 27.29 | 1.22 |
| hsa-miR-34a-5p | 25.56 | 0.38 |
| hsa-miR-34b-3p | 30.65 | 0.04 |
| hsa-miR-92a-3p | 24.99 | 0.14 |
| hsa-miR-93-3p | 28.48 | 0.51 |
| hsa-miR-93-5p | 25.47 | 0.19 |
| hsa-miR-95-3p | 30.16 | 2.96 |
| hsa-miR-99a-3p | 33.20 | 0.06 |
| hsa-miR-99a-5p | 29.28 | 0.73 |
| hsa-miR-99b-3p | 28.15 | 0.10 |
| hsa-miR-99b-5p | 27.59 | 0.33 |
| hsa-miR-100-5p | 30.12 | 0.48 |
| hsa-miR-101-3p | 31.98 | 1.04 |
| hsa-miR-103a-3p | 28.13 | 0.23 |
| hsa-miR-106a-5p | 20.77 | 0.79 |
| hsa-miR-106b-5p | 25.13 | 0.48 |
| hsa-miR-125a-3p | 30.64 | 0.31 |
| hsa-miR-125a-5p | 24.32 | 0.10 |
| hsa-miR-125b | 29.23 | 0.80 |
| hsa-miR-126-3p | 23.40 | 0.87 |
| hsa-miR-126-5p | 27.94 | 1.42 |
| hsa-miR-128 | 29.84 | 0.17 |
| hsa-miR-130a-3p | 29.03 | 0.52 |
| hsa-miR-130b-3p | 29.22 | 0.91 |
| hsa-miR-132-3p | 25.43 | 0.16 |
| hsa-miR-133a-3p | 30.39 | 1.19 |
| hsa-miR-135a-5p | 30.86 | 0.44 |
| hsa-miR-135b-5p | 24.12 | 0.12 |
| hsa-miR-136-5p | 31.74 | 0.48 |
| hsa-miR-137 | 30.22 | 2.26 |
| hsa-miR-138-5p | 21.82 | 1.90 |
| hsa-miR-139-5p | 26.82 | 0.43 |
| hsa-miR-140-3p | 30.88 | 0.58 |
| hsa-miR-140-5p | 24.61 | 0.01 |
| hsa-miR-141-3p | 32.75 | 0.49 |
| hsa-miR-142-3p | 28.54 | 1.06 |
| hsa-miR-143-3p | 31.62 | 0.32 |
| hsa-miR-145-5p | 29.84 | 0.89 |
| hsa-miR-146a-5p | 15.86 | 0.31 |
| hsa-miR-146b-3p | 33.01 | 0.43 |
| hsa-miR-146b-5p | 20.93 | 0.06 |
| hsa-miR-148a-3p | 28.91 | 0.41 |
| hsa-miR-148b-3p | 31.84 | 0.49 |
| hsa-miR-148b-5p | 31.87 | 0.60 |
| hsa-miR-149-5p | 28.14 | 1.02 |
| hsa-miR-150-5p | 23.70 | 1.48 |
| hsa-miR-151a-3p | 24.62 | 0.12 |
| hsa-miR-151a-5p | 29.93 | 0.03 |
| hsa-miR-152 | 25.87 | 0.19 |
| hsa-miR-155-5p | 18.68 | 0.93 |
| hsa-miR-181a-5p | 27.15 | 0.15 |
| hsa-miR-181c-5p | 32.73 | 0.41 |
| hsa-miR-182-5p | 27.67 | 0.42 |
| hsa-miR-181a-2-3p | 30.40 | 1.15 |
| hsa-miR-183-3p | 30.45 | 0.58 |
| hsa-miR-184 | 32.90 | 0.96 |
| hsa-miR-186-5p | 21.82 | 0.27 |
| hsa-miR-190b | 32.84 | 1.25 |
| hsa-miR-191-3p | 33.24 | 0.48 |
| hsa-miR-191-5p | 19.67 | 0.14 |
| hsa-miR-192-3p | 31.56 | 0.04 |
| hsa-miR-192-5p | 25.56 | 0.73 |
| hsa-miR-193a-5p | 27.94 | 0.03 |
| hsa-miR-193b-3p | 21.03 | 0.07 |
| hsa-miR-194-5p | 25.07 | 0.63 |
| hsa-miR-195-5p | 26.43 | 0.99 |
| hsa-miR-196b-5p | 24.53 | 0.35 |
| hsa-miR-197-3p | 27.17 | 0.37 |
| hsa-miR-199a-3p | 30.87 | 1.23 |
| hsa-miR-200a-3p | 24.13 | 0.04 |
| hsa-miR-200a-5p | 28.56 | 0.22 |
| hsa-miR-200b-3p | 22.87 | 0.21 |
| hsa-miR-200c-3p | 29.82 | 3.46 |
| hsa-miR-203 | 28.82 | 0.45 |
| hsa-miR-204-5p | 20.26 | 0.01 |
| hsa-miR-210 | 24.28 | 0.64 |
| hsa-miR-212-3p | 31.22 | 0.76 |
| hsa-miR-215-5p | 31.08 | 3.51 |
| hsa-miR-218-5p | 21.14 | 0.03 |
| hsa-miR-221-3p | 25.02 | 0.48 |
| hsa-miR-222-3p | 19.68 | 0.62 |
| hsa-miR-222-5p | 30.77 | 1.59 |
| hsa-miR-223-3p | 21.91 | 1.72 |
| hsa-miR-224-5p | 27.00 | 0.16 |
| hsa-miR-296-5p | 31.42 | 0.20 |
| hsa-miR-301a-3p | 28.95 | 0.82 |
| hsa-miR-302a-3p | 21.24 | 0.29 |
| hsa-miR-320a | 21.79 | 0.24 |
| hsa-miR-324-3p | 26.46 | 0.90 |
| hsa-miR-324-5p | 29.83 | 1.30 |
| hsa-miR-326 | 32.66 | 0.36 |
| hsa-miR-328 | 28.19 | 1.26 |
| hsa-miR-330-3p | 31.32 | 0.57 |
| hsa-miR-331-3p | 23.30 | 0.67 |
| hsa-miR-331-5p | 30.48 | 1.87 |
| hsa-miR-335-5p | 32.55 | 0.70 |
| hsa-miR-339-3p | 27.38 | 0.13 |
| hsa-miR-339-5p | 32.88 | 0.56 |
| hsa-miR-340-5p | 28.21 | 0.46 |
| hsa-miR-340-3p | 32.72 | 1.36 |
| hsa-miR-342-3p | 28.59 | 0.75 |
| hsa-miR-342-5p | 22.85 | 0.21 |
| hsa-miR-345-5p | 26.33 | 0.32 |
| hsa-miR-361-5p | 29.28 | 0.23 |
| hsa-miR-362-3p | 32.47 | 1.24 |
| hsa-miR-362-5p | 29.43 | 0.31 |
| hsa-miR-374a-5p | 23.88 | 0.07 |
| hsa-miR-374b-5p | 21.98 | 0.23 |
| hsa-miR-375 | 32.71 | 0.09 |
| hsa-miR-376a-3p | 30.52 | 1.63 |
| hsa-miR-376b-3p | 30.34 | 2.10 |
| hsa-miR-376c | 28.56 | 1.17 |
| hsa-miR-378a-3p | 28.58 | 0.11 |
| hsa-miR-409-3p | 30.09 | 3.61 |
| hsa-miR-410-3p | 31.14 | 0.71 |
| hsa-miR-411-5p | 28.57 | 0.76 |
| hsa-miR-422a | 24.82 | 0.19 |
| hsa-miR-423-5p | 30.45 | 0.25 |
| hsa-miR-425-3p | 29.77 | 1.27 |
| hsa-miR-425-5p | 27.74 | 0.59 |
| hsa-miR-429 | 24.63 | 0.32 |
| hsa-miR-432-5p | 30.61 | 1.94 |
| hsa-miR-449a | 30.66 | 1.82 |
| hsa-miR-451a | 28.60 | 1.02 |
| hsa-miR-452-5p | 27.67 | 0.52 |
| hsa-miR-454-3p | 23.63 | 0.20 |
| hsa-miR-455-3p | 32.01 | 2.02 |
| hsa-miR-455-5p | 28.30 | 0.54 |
| hsa-miR-483-5p | 29.36 | 1.74 |
| hsa-miR-484 | 22.54 | 0.40 |
| hsa-miR-487b-3p | 31.07 | 1.78 |
| hsa-miR-491-5p | 31.76 | 0.71 |
| hsa-miR-495-3p | 31.95 | 2.57 |
| hsa-miR-500a-5p | 30.75 | 0.88 |
| hsa-miR-501-5p | 30.94 | 0.75 |
| hsa-miR-502-5p | 33.42 | 1.06 |
| hsa-miR-503 | 26.45 | 0.32 |
| hsa-miR-505-5p | 32.80 | 0.86 |
| hsa-miR-518b | 29.20 | 0.26 |
| hsa-miR-518f-3p | 17.78 | 1.93 |
| hsa-miR-519a-3p | 31.98 | 2.18 |
| hsa-miR-520a-3p | 24.57 | 0.75 |
| hsa-miR-523-3p | 21.00 | 1.46 |
| hsa-miR-532-3p | 27.00 | 0.13 |
| hsa-miR-532-5p | 25.98 | 0.15 |
| hsa-miR-539-5p | 31.04 | 2.11 |
| hsa-miR-545-3p | 33.01 | 1.14 |
| hsa-miR-548b-3p | 26.31 | 0.27 |
| hsa-miR-561-3p | 22.61 | 1.00 |
| hsa-miR-574-3p | 22.56 | 0.52 |
| hsa-miR-577 | 29.66 | 0.12 |
| hsa-miR-589-3p | 33.58 | 0.27 |
| hsa-miR-590-3p | 28.53 | 0.08 |
| hsa-miR-590-5p | 24.25 | 1.01 |
| hsa-miR-597-5p | 31.78 | 0.78 |
| hsa-miR-598 | 27.59 | 0.10 |
| hsa-miR-605-5p | 33.43 | 0.01 |
| hsa-miR-618 | 27.48 | 3.67 |
| hsa-miR-625-3p | 26.48 | 0.38 |
| hsa-miR-628-3p | 30.93 | 0.20 |
| hsa-miR-628-5p | 26.80 | 0.28 |
| hsa-miR-629-3p | 29.89 | 0.05 |
| hsa-miR-636 | 33.16 | 1.76 |
| hsa-miR-642a-5p | 24.69 | 0.91 |
| hsa-miR-652-3p | 30.90 | 0.67 |
| hsa-miR-660-5p | 26.54 | 0.21 |
| hsa-miR-664a-3p | 28.43 | 2.53 |
| hsa-miR-671-3p | 31.79 | 0.34 |
| hsa-miR-708-5p | 23.52 | 0.29 |
| hsa-miR-720 | 19.57 | 0.30 |
| hsa-miR-744-5p | 27.01 | 0.69 |
| hsa-miR-766-3p | 30.32 | 0.89 |
| hsa-miR-885-5p | 29.92 | 1.17 |
| hsa-miR-886-3p | 25.57 | 0.87 |
| hsa-miR-886-5p | 24.48 | 1.45 |
| hsa-miR-942-5p | 33.96 | 0.23 |
| hsa-miR-1180-3p | 33.23 | 0.46 |
| hsa-miR-1226-5p | 31.51 | 1.24 |
| hsa-miR-1227-3p | 32.40 | 1.53 |
| hsa-miR-1233-3p | 30.81 | 1.14 |
| hsa-miR-1243 | 30.69 | 1.07 |
| hsa-miR-1260a | 27.60 | 1.00 |
| hsa-miR-1269a | 32.21 | 0.59 |
| hsa-miR-1271-5p | 29.42 | 0.57 |
| hsa-miR-1274A | 20.06 | 1.06 |
| hsa-miR-1274B | 18.60 | 0.92 |
| hsa-miR-1290 | 24.61 | 0.74 |
| hsa-miR-1291 | 32.13 | 0.62 |
| hsa-miR-1300 | 30.40 | 0.27 |
| hsa-miR-1305 | 18.20 | 0.66 |
