# Supplementary material for: Mesenchymal Stromal Cells Epithelial Transition Induced by Renal Tubular Cells-Derived Extracellular Vesicles
Source: PLoS One. 2016 Jul 13;11(7):e0159163. doi: 10.1371/journal.pone.0159163 (PMC4943710; doi:10.1371/journal.pone.0159163)
Supplement: S2 Table — (DOCX) [file pone.0159163.s004.docx]

| **KEGG pathway** | **p-value** | **#genes** | **#miRNAs** |
| --- | --- | --- | --- |
| Endocytosis (hsa04144) | <1e-16 | 33 | 2 |
| Protein digestion and absorption (hsa04974) | <1e-16 | 24 | 2 |
| Endometrial cancer (hsa05213) | <1e-16 | 24 | 3 |
| Small cell lung cancer (hsa05222) | <1e-16 | 25 | 3 |
| Glycosaminoglycan biosynthesis - chondroitin sulfate (hsa00532) | <1e-16 | 6 | 4 |
| ECM-receptor interaction (hsa04512) | <1e-16 | 20 | 4 |
| Insulin signaling pathway (hsa04910) | <1e-16 | 35 | 4 |
| Melanoma (hsa05218) | <1e-16 | 32 | 4 |
| Wnt signaling pathway (hsa04310) | <1e-16 | 66 | 5 |
| Gap junction (hsa04540) | <1e-16 | 30 | 5 |
| Long-term potentiation (hsa04720) | <1e-16 | 34 | 5 |
| Amoebiasis (hsa05146) | <1e-16 | 37 | 5 |
| Transcriptional misregulation in cancer (hsa05202) | <1e-16 | 60 | 5 |
| Renal cell carcinoma (hsa05211) | <1e-16 | 32 | 5 |
| Glioma (hsa05214) | <1e-16 | 33 | 5 |
| ErbB signaling pathway (hsa04012) | <1e-16 | 35 | 6 |
| mTOR signaling pathway (hsa04150) | <1e-16 | 28 | 6 |
| Hepatitis B (hsa05161) | <1e-16 | 49 | 6 |
| Ubiquitin mediated proteolysis (hsa04120) | <1e-16 | 58 | 7 |
| TGF-beta signaling pathway (hsa04350) | <1e-16 | 41 | 7 |
| Pathways in cancer (hsa05200) | <1e-16 | 126 | 7 |
| Prostate cancer (hsa05215) | <1e-16 | 49 | 7 |
| Chronic myeloid leukemia (hsa05220) | <1e-16 | 32 | 7 |
| MAPK signaling pathway (hsa04010) | <1e-16 | 84 | 8 |
| p53 signaling pathway (hsa04115) | <1e-16 | 33 | 8 |
| Axon guidance (hsa04360) | <1e-16 | 56 | 8 |
| Focal adhesion (hsa04510) | <1e-16 | 94 | 8 |
| Neurotrophin signaling pathway (hsa04722) | <1e-16 | 48 | 8 |
| PI3K-Akt signaling pathway (hsa04151) | <1e-16 | 159 | 12 |
| Colorectal cancer (hsa05210) | 3.330669e-16 | 27 | 5 |
| Non-small cell lung cancer (hsa05223) | 3.330669e-16 | 25 | 5 |
| Fatty acid biosynthesis (hsa00061) | 8.881784e-16 | 2 | 1 |
| Pancreatic cancer (hsa05212) | 9.325873e-15 | 28 | 3 |
| Lysine degradation (hsa00310) | 4.551914e-14 | 14 | 4 |
| Acute myeloid leukemia (hsa05221) | 4.196643e-13 | 19 | 3 |
| Regulation of actin cytoskeleton (hsa04810) | 3.585687e-12 | 20 | 1 |
| Dopaminergic synapse (hsa04728) | 2.022116e-11 | 28 | 1 |
| HTLV-I infection (hsa05166) | 7.799994e-11 | 25 | 1 |
| GnRH signaling pathway (hsa04912) | 3.385604e-10 | 25 | 3 |
| Protein processing in endoplasmic reticulum (hsa04141) | 9.902995e-10 | 31 | 2 |
| Melanogenesis (hsa04916) | 2.450122e-09 | 41 | 3 |
| Progesterone-mediated oocyte maturation (hsa04914) | 1.73783e-08 | 25 | 4 |
| Hepatitis C (hsa05160) | 2.299145e-08 | 17 | 3 |
| HIF-1 signaling pathway (hsa04066) | 5.076362e-08 | 17 | 1 |
| Circadian rhythm (hsa04710) | 7.311481e-08 | 10 | 2 |
| Glutamatergic synapse (hsa04724) | 6.893008e-07 | 28 | 3 |
| RNA degradation (hsa03018) | 1.510346e-06 | 19 | 2 |
| Aldosterone-regulated sodium reabsorption (hsa04960) | 2.054241e-06 | 16 | 3 |
| Hypertrophic cardiomyopathy (HCM) (hsa05410) | 2.638966e-06 | 18 | 3 |
| Gastric acid secretion (hsa04971) | 7.556783e-06 | 24 | 3 |
| Basal cell carcinoma (hsa05217) | 1.214242e-05 | 9 | 1 |
| Hedgehog signaling pathway (hsa04340) | 1.950418e-05 | 15 | 2 |
| Long-term depression (hsa04730) | 6.850153e-05 | 17 | 2 |
| Sphingolipid metabolism (hsa00600) | 8.782205e-05 | 7 | 2 |
| B cell receptor signaling pathway (hsa04662) | 9.886762e-05 | 12 | 1 |
| Viral carcinogenesis (hsa05203) | 0.0001058329 | 34 | 2 |
| Chemokine signaling pathway (hsa04062) | 0.000160481 | 13 | 1 |
| Oocyte meiosis (hsa04114) | 0.0002067098 | 30 | 2 |
| Salmonella infection (hsa05132) | 0.0002457006 | 9 | 2 |
| Arrhythmogenic right ventricular cardiomyopathy (ARVC) (hsa05412) | 0.0002624394 | 16 | 2 |
| Shigellosis (hsa05131) | 0.0002979481 | 7 | 1 |
| Cholinergic synapse (hsa04725) | 0.000468587 | 20 | 1 |
| Endocrine and other factor-regulated calcium reabsorption (hsa04961) | 0.0007509536 | 10 | 1 |
